# Supplementary material for: Quantification and discovery of sequence determinants of protein‐per‐mRNA amount in 29 human tissues
Source: Mol Syst Biol. 2019 Feb 18;15(2):e8513. doi: 10.15252/msb.20188513 (PMC6379048; doi:10.15252/msb.20188513)
Supplement: Supplementary file 1 — Appendix [file MSB-15-e8513-s001.pdf]

Appendix to the manuscript “Quantification and discovery of sequence determinants of protein per mRNA amount in 29 human tissues”

Basak Eraslan [1, 2,#], Dongxue Wang [3, #]
Mirjana Gusic [5,6], Holger Prokisch [5,6]
Björn Hallström [7], Mathias Uhlen [7]
Anna Asplund [8], Frederik Ponten [8]
Thomas Wieland [3,] Thomas Hopf [3]
Hannes Hahne [4,*]
Bernhard Kuster [3, 9, *]
Julien Gagneur[1,*]

1 Computational Biology, Department of Informatics, Technical University of Munich, Boltzmannstr. 3, 85748, Garching bei München, Germany
2 Graduate School of Quantitative Biosciences (QBM), Ludwig-Maximilians-Universität München, Germany
3 Chair of Proteomics and Bioanalytics, Technische Universität München, Emil-Erlenmeyer-Forum 5, 85354 Freising, Germany
4 OmicScouts GmbH, Lise-Meitner-Str. 30, 85354 Freising, Germany
5 Institute of Human Genetics, Technical University of Munich, 81675 München, Germany
6 Institute of Human Genetics, Helmholtz Zentrum München, 85764 Neuherberg, Germany
7 Science for Life Laboratory, KTH - Royal Institute of Technology, Stockholm, Sweden
8 Department of Immunology, Genetics and Pathology, Science for Life Laboratory, Uppsala University, Uppsala, Sweden
9 Center For Integrated Protein Science Munich (CIPSM), Munich, Germany

# Contributed equally
* Corresponding authors

Table of Contents

DESCRIPTION OF 5'UTR MOTIFS FOUND DE NOVO..2
DESCRIPTION OF 3'UTR MOTIFS FOUND DE NOVO..6
SUPPLEMENTARY FIGURES 10
REFERENCES 30

Description of 5'UTR motifs found de novo

- AACUU, was present in 5' UTR of 1,390 (12%) of the 11,575 investigated genes, associated with 12% decrease in PTR ratio only in brain (Figure 2E). According to the current knowledge of RBP binding motifs, AACUU may be bound by Serine/arginine-rich splicing factor 3 (SRSF3) with low probability (Qscore 0.002). AACUU sites were significantly evolutionarily conserved (P-value : 1.63e-16) compared to the background flanking regions (Appendix Figure S5A). The genes that contains the consensus sequence of AACUU were enriched in pri-miRNA transcription from RNA polymerase 2 promoter biological process (Appendix Figure S6A) and transcription factor activity metabolic function (Appendix Fig S5B).

- ACCUGC, present in 886 genes (8%), associated with 15% less PTR ratio in stomach only (Figure 2E) and is similar to the consensus target sequence of mRNA decay activator protein ZFP36 (Qscore 0.03). ACCUUGC was significantly evolutionarily conserved (P-value: 3.79e-10, Appendix Figure S5B) and enriched for genes in ion transport biological process (Appendix Figure S6C) and localized in plasma membrane (Appendix Figure S6D).

- AGCAAC, present in 524 genes (5%), on average associated with 20% less PTR ratio in ovary and prostate (Figure 2E) and was enriched for genes of cilium morphogenesis (Appendix Figure S6E) which were localized in cilium (Appendix Figure S6F).

- AGCCCCC, present in 499 genes (4%), associated with 20% less PTR ratio in kidney and liver (Figure 2E). AGCCCCC was significantly conserved through evolution (P-

value:2.15e-07, Appendix Figure S5D) and was enriched for genes in various biological processes (Appendix Figure S6G), localized in cell leading edge (Appendix Figure S6H) and recruiting kinase binding metabolic function (Appendix Figure S6I).

- AGCGGAA, present in 215 genes (2%), on average associated with 30% less PTR ratio in appendix, esophagus, placenta, prostate, and rectum (Figure 2E). AGCGGAA was significantly conserved (P-value: 2.37e-24, Appendix Figure S5E) and enriched for genes localized in nuclear part (Appendix Figure S6J).

- CACGU, present in 1,064 genes (9%), on average associated with 15% PTR increase in colon, duodenum, endometrium, esophagus, liver, small intestine, testis and 28% increase in lung. CACGU may be the target site of Serine/Argenine-rich splicing factor 1 (SRSF1) with low probability (Qscore 0.03). CACGU was highly conserved through evolution (P-value : 2.81e-48, Appendix Figure S5F) and enriched in genes active in ribosome biogenesis (Appendix Figure S6K) localized in nucleolus (Appendix Figure S6L).

- CAGAC, present in 2,352 genes (20%), associated with 12% less PTR ratio only in pancreas (Figure 2E). CAGAC matches the binding motifs of several RBPs, such as HNRLPLL (Qscore 1.0), SF1 (Qscore 1.0), SRSF1 (Qscore 1.0) which are important factors of splicing and ENOX1 (Qscore 0.09), which plays a role in control of the ultradian cellular biological clock (Wang *et al*, 2003). The genes enriched in CAGAC were localized in intrinsic component of membrane (Appendix Figure S6M).

- CCCACCC, present in 716 genes (6%), significantly associated with 20% higher PTR ratio in lymph node. CCCACCC matches with the target motifs of PCBP1 (Qscore 1.0), poly(rc) binding protein 1, and G3BP1 (Qsore 0.01). The two C triplets appear to be important in this motif because mismatches are rare in the genome (Figure 2F) and because these positions were more conserved than the central nucleotide and flanking nucleotides (Pval: 1.93e-07, Appendix Figure S5H).

- CCGUGGG, present in 289 genes (2%), significantly associated with 30% less PTR ratio only in appendix (Figure 2E). CCGUGGG vaguely resembles the target motif of SRFS1 (Qscore 0.007).

- CCUUGGA, present in 260 genes (2%), is significantly associated with over 40% PTR ratio increase only in liver and placenta (Figure 2E).

- CGGAAG, present in 1,131 genes (10%), significantly associated with 14% higher PTR ratio in lung and urinary bladder (Figure 2E). CGGAAG matches the binding target of SFRSF1. Consensus motif sites of CGGAAG were highly conserved through evolution (Appendix Figure S5K) and the genes containing these sites were enriched in specific biological processes (Appendix Figure S6N) localized in certain cell components (Appendix Figure S6O).

- CUCUGAG, present in 399 genes (3%), significantly associated with 30% higher PTR ratio only in prostate (Figure 2E).

- CUCUUUC, present in 311 genes (3%), significantly associated with 20% less PTR ratio only in prostate (Figure 2E).

- CUGGGAGC, present in 253 genes (2%), significantly associated with 25% less PTR ratio only in rectum (Figure 2E).

- CUGUCCU, present in 323 genes (3%), on average associated with 41% PTR ratio in 18 tissues (Figure 2E). The genes with the consensus sequence of this motif were enriched in multicellular organismal process (Appendix Figure S6P).

- GAUAC, present in 587 genes (5%), associated with 20% less PTR ratio in testis (Figure 2E). GAUAC is the recognition target of HNRNPC (Qscore 1.0), which is highly expressed in testis (FANTOM5 data (Lizio *et al*, 2015; FANTOM Consortium and the RIKEN PMI and CLST (DGT) *et al*, 2014)), and modulates the stability and the level of translation of bound mRNA molecules (Kanhoush *et al*, 2010; Shetty, 2005).

- GCCGCC, present in 3,038 genes (26%), significantly associated with 5% less PTR ratio in ten tissues (Figure 2E). GCCGCC matches with the recognition motif of RBP SRSF2 (Qscore 0.24), and the genes having the consensus GCCGCC sequence are enriched in various Gene Ontology terms (Appendix Figure S6Q, S5R, S5S).

- GGCGCCCG, present in 413 genes (4%), on average associated with 30% less PTR ratio in twenty-four tissues (Figure 2E).
- GUGGGAA, present in 261 genes (2%), associated with 50% higher PTR ratio in liver (Figure 2E). GUGGGAA consensus motif sites were highly conserved (P-value: 1.69e-06, Appendix Figure S5S) through evolution.
- UACAGG, present in 331 genes (3%), associated with 26% higher PTR ratio in stomach (Figure 2E) and matches with the target site of SRSF6 with Qscore being equal to 0.03.
- UCGAC, present in 662 genes (6%), associated with 20% less PTR ratio in duodenum and ovary (Figure 2E). Consensus sequence of UCGAC mildly matches with the target motif of SRSF3 (Qscore 0.03) and the genes having this motif were enriched in axon regeneration process (Appendix Figure S6T).
- UCUGGGA, present in 382 genes (3%), associated with 30% less PTR ratio in adrenal gland (Figure 2E). UCUGGGA is possibly bound by GRSF1, regulator of post-transcriptional mitochondrial gene expression (Antonicka *et al*, 2013), also comparatively highly expressed in adrenal gland (Human Protein Atlas, (Uhlen *et al*, 2015)). Even though the conservation of the whole consensus sequence is not significant (P-value: 6.72e-02, Appendix Figure S5V), the last four nucleotides of the motif, namely GGGA, was significantly evolutionarily conserved (P-value: 1.12e-08) compared to the flanking regions.
- UGACCU, present in 557 genes (5%), associated with 23% less PTR ratio in duodenum (Figure 2E). Consensus motif sites of UGACCU were highly conserved (P-value: 7.41e-19, Appendix Figure S5W).
- UUCCG, present in 2,182 genes (19%), associated with 10% higher PTR ratio in lung and thyroid (Figure 2E). UUCCG may be bound by SRSF2 with low probability (Qscore 0.002). Consensus motif sites of UUCCG were highly conserved through evolution (Appendix Figure S5X) and the genes that had the consensus sequence were significantly enriched in several biological processes such as cellular component biogenesis, RNA export from nucleus, organitrogen compound biosynthesis and intracellular transport processes (Appendix Figure S6U) and were localized in membrane-bounded organelles (Appendix Figure S6V).

Description of 3'UTR motifs found de novo

- AAUAAA, one of the recovered known mRNA motifs in 3' UTR, is the polyadenylation signal (Proudfoot, 1991). AAUAAA associated with between 13% and 28% increased PTR ratio across twenty-three tissues (median 21%, <0.1 FDR, Figure 5A), consistent with a role of polyadenylation signals in translation (Piqué *et al*, 2008).
- ACAAGUC, present in 623 genes (5%), associated with 20% less PTR ratio only in spleen.
- ACACUA, present in 2,175 genes (19%), on average associated with 10% higher PTR ratio in adrenal gland, appendix, brain and fat (Figure 5A). ACACUA is the core sequence of the target motif of RNA binding protein QKI (Qscore 1.0), which is highly enriched in brain (Human Protein Atlas, (Uhlen *et al*, 2015)) and important for myelinization (Aberg *et al*, 2006), mRNA stability and protein translation (Teplova *et al*, 2013). Consistent with the function of QKI, the genes that contain the consensus sequence ACACUA were enriched in signal transduction process (Appendix Figure S12A).
- ACCAAA, present in 3,655 genes (32%), on average associated with 7% higher PTR ratio in adrenal gland, brain, pancreas, placenta, rectum and urinary bladder (Figure 5A). ACCAAA is the perfect match of the target motif of RBMX (Qscore 1.0) which plays several roles in the regulation of post-transcriptional processes (Kanhoush *et al*, 2010). Consensus motif sites of ACCAAA in 3' UTR are highly conserved (P-value: 9.08e-112, Appendix Figure S11D) and the genes containing these sites are enriched in cell communication, signaling processes (Appendix Figure S12C) which are intrinsic components of the membrane (Appendix Figure S12D).
- ACUCUG, present in 3,827 genes (33%), associated with 6% higher PTR ratio in duodenum, endometrium, fallopian tube, heart, liver, lung, lymph node, placenta, prostate, spleen and testis (Figure 5A). The genes containing the consensus ACUCUG sequence were enriched in glycoprotein metabolic process (Appendix Figure S12E) and were components of membrane and cell periphery (Appendix Figure S12F).
- AUGAGAC, present in 805 genes (7%), associated with 24% higher PTR ratio in gallbladder. Genes with the consensus motif were enriched in cell communication process (Appendix Figure S12G).
- AUUUUUA, present in 4,119 (36%) genes, is another recovered well known AU-rich element (ARE) (Chung *et al*, 1996). AUUUUUA on average associated with 5% higher PTR ratio in fifteen tissues (Figure 5A). There are several ARE-binding proteins including

HNRNPD, Hu proteins (ELAV family), ZFP36 and TIAL1 and the motif sites of this ARE are highly conserved (Appendix Figure S11G) through evolution.

- CAACAGA, present in 381 genes (3%), associated between 22% and 30% decrease in PTR ratio in fourteen tissues (Figure 5A).
- CCAAAG, present in 3,992 (34%) genes, associated with 6% higher PTR ratio in fallopian tube, lymph node, small intestine and tonsil. Binding target proteins of CCAAAG is not reported before but its big number of occurrences and highly conserved motif sites in 3' UTR sequences (P-value: 1.08e-149, Appendix Figure S11I) signal that it may be a key regulatory motif. The genes containing CCAAAG are enriched in signaling process (Appendix Figure S12J), localized in membrane (Appendix Figure S12K).
- CCUGUA, present in 3,484 genes (30%), on average associated with 5% increased PTR ratio in seventeen tissues (Figure 5A). CCUGUA matches with the recognition motif of signal recognition particle 14 kDa protein SRP14 (Qscore 1.0). The genes containing the consensus sequence of CCUGUA are enriched in bone development (Appendix Figure S12L) and magnesium ion binding (Appendix Figure S12M).
- CGUGUGG, present in 380 genes (3%), associated with 38% higher PTR in esophagus (Figure 5A). Consistent with the information content, the conservation of the first two nucleotides of this motif is much smaller compared to UGUGG (Appendix Figure S11K). Even though not any of the annotated RBPs recognize the full CGUGUGG, the subsequence, UGUGG, is part of the recognition motifs of several RBPs including HNRNPF (Qscore 1.0), HNRNPH family proteins (Qscore 1.0), SRSF6 (Qscore 1.0) and ZFP36 (Qscore 0.49).
- CUCAGG, present in 3,980 genes (34%), associated with 6% less PTR ratio in testis. CUCAGG slightly matches the recognition motif of SRSF6 (Qscore 0.03). It is highly conserved through evolution (P-value 1.16e-136, Appendix Figure S11L) and the genes containing this motif in their 3' UTR sequences are enriched in small GTPase mediated signal transduction process (Appendix Figure S12N).
- GGGCUGCG, present in 169 genes (1%), on average associated with 30% less PTR ratio in colon, esophagus, fallopian tube, fat, gallbladder, salivary gland and urinary bladder (Figure 5A). First six nucleotides, GGGCUG, match with the recognition motifs of HNRNPF(Qscore 1.0), HNRNPH1(Qscore 1.0), HNRNPH2(Qscore 1.0) and HNRNPH3(Qscore 1.0). GGGCUGCG is significantly conserved compared to flanking regions (P-value: 2.52e-02, Appendix Figure S11M).

- GGAGCC, present in 3,140 genes (27%), on average associated with 4% less PTR ratio in fat, kidney, lymph node, ovary and thyroid (Figure 5A). GGAGCC matches with the recognition targets of three heterogeneous nuclear ribonucleoprotein (hnRNP) protein family members, namely HNRNPA1 (Qscore 1.0), HNRNPA2B1 (Qscore 1.0), and HNRNPA3 (Qscore 1.0). These RBPs has multiple roles including mRNA stabilization, and translational regulation (Geuens *et al*, 2016) and they are highly expressed in lymph node, ovary and thyroid gland consistent with their effect significance. Consensus motif sites of GGAGCC are highly conserved in 3’ UTR (P-value 8.66e-106, Appendix Figure S11N) and the genes containing this consensus sequence are significantly enriched in various regulatory biological processes (Appendix Figure S12P) localized in cell periphery (Appendix Figure S12Q).
- GGCCCCUG, present in 571 genes (5%), on average associated with 23% higher PTR ratio in adrenal gland, brain, endometrium, heart, liver and thyroid (Figure 5A). Consensus sequence matches with the target motif of SRSF2 (Qscore 0.02) and the motif sites of the consensus sequence is significantly conserved compared to flanking regions (P-value 2.54e-03, Appendix Figure S11O). Genes with the consensus GGCCCCUG sequence in their 3’ UTR regions are enriched in regulation of signal transduction (Appendix Figure S12S).
- UACUAAGA, present in 222 genes (2%), on average associated with 43% higher PTR ratio (min 32%, max 65%) in twenty-three tissues (Figure 5A).
- UAUGCA, present in 2,858 genes (25%), associated with 8% higher PTR ratio in appendix and colon (Figure 5A). Genes having the consensus sequence were enriched in intracellular signal transduction process (Appendix Figure S12T) and metal ion transmembrane transporter activity (Appendix Figure S12V).
- UAUUUAU, another recovered AU-rich element was present in 3,158 genes (27%), and on average associated with 10% less PTR ratio in all tissues (Figure 5A). The consensus motif sites of UAUUUAU are highly conserved in 3’ UTR (Appendix Figure S11R) and the genes having this motif are enriched in various biological processes (Appendix Figure S12 W), especially localized in Golgi apparatus (Appendix Figure S12Y).
- UGUAAAUA, present in 1,320 (11%) genes, was another recovered well known motif bound by the Pumilio family of proteins (Filipovska *et al*, 2011) which act as a post-transcriptional repressor (Parisi & Lin, 2000). Consistent with the function of the bound protein, this motif on average associated with 15% less PTR ratio in twenty two tissues (Figure 5A). Motif sites of UGUAAAUA were highly conserved through evolution (P-value

6.18e-285, Appendix Figure S11S) and the genes containing this motif were enriched in various biological processes (Appendix Figure S12Z), mostly localized in nuclear lumen (Appendix Figure S12A1).

- UUCUGAG, present in 1,818 (16%) genes, associated with 11% higher PTR ratio in appendix, fat and small intestine (Figure 5A). Even though binding proteins recognizing this motif was not available in RBP databases, very high conservation score of the motif (P-value 1.24e-64, Appendix Figure S11T) suggests this motif to be a candidate motif important for post-transcriptional regulation. The genes containing the consensus sequence UUCUGAG in their 3' UTR sites were enriched in protein ubiquitination process (Appendix Figure S12C2).

Supplementary Figures

S1

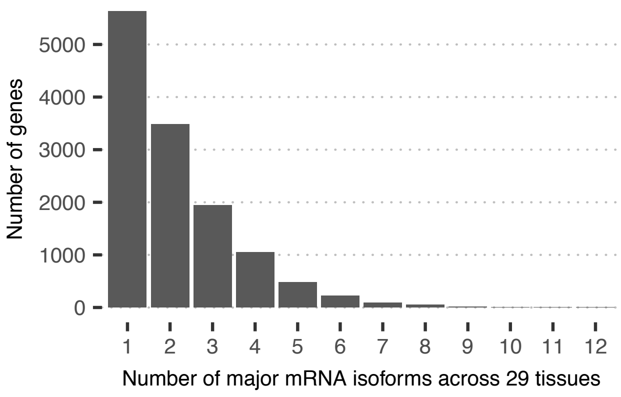

Figure S1: Distribution of the number of different major mRNA isoforms each gene has across 29 tissues.

S2

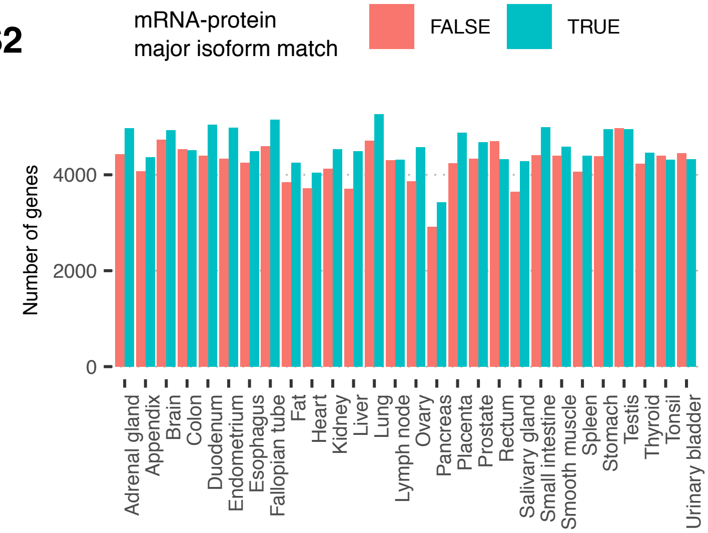

Figure S2: Number of genes in each tissue with matched and unmatched mRNA and protein major isoforms.

S3

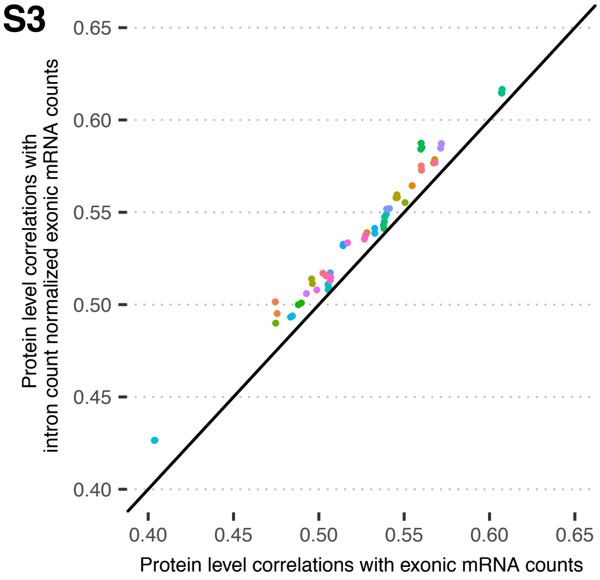

Figure S3: Spearman's correlation coefficients between tissue-specific proteome and transcriptome with (y-axis, Materials and Methods) and without intronic reads normalization (x-axis).

S4

A

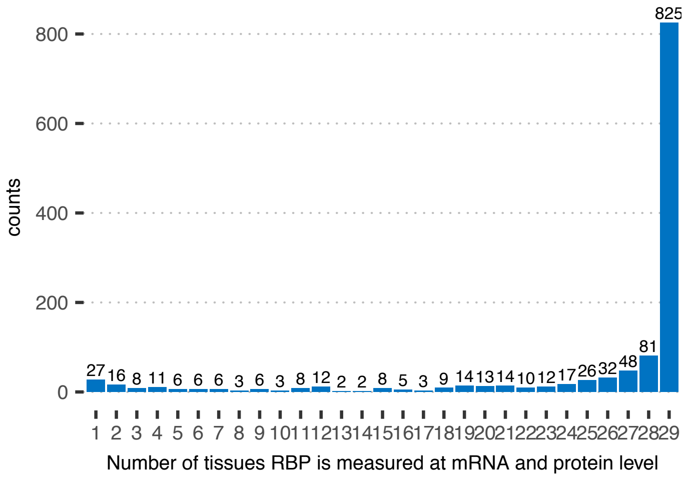

Figure S4-A: Distribution of the number of tissues 1,233 RNA binding proteins are measured both at transcriptome and proteome level.

B

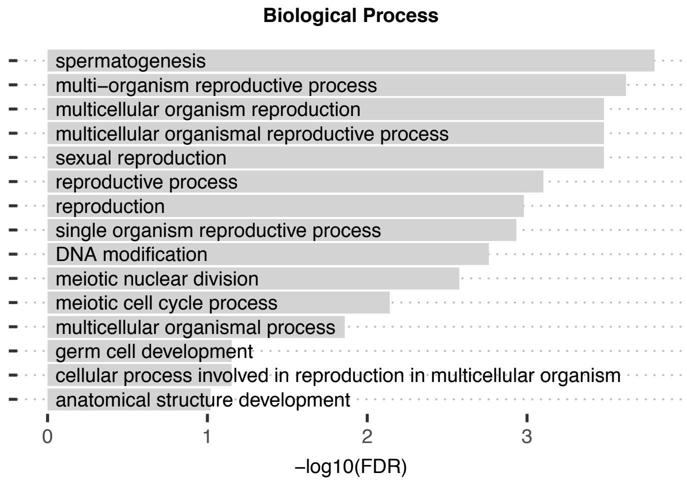

Figure S4-B: Gene ontology terms (biological process) that are enriched for 135 genes that are found to be tissue-specific according to mRNA tissue-specificity scores defined by Gerstberger et al.

C

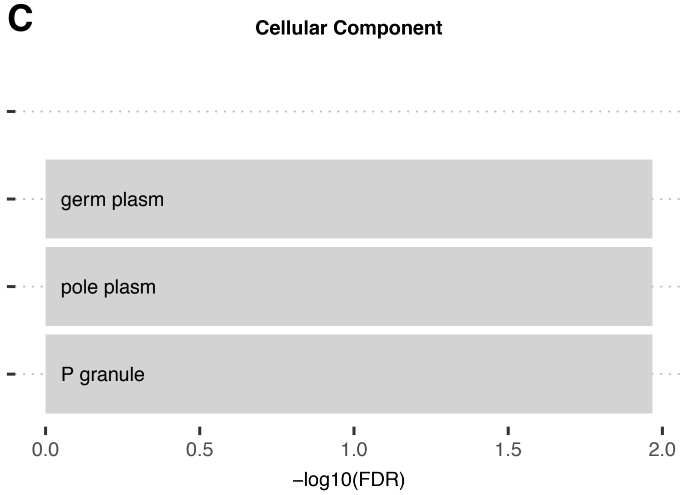

Figure S4-C: Similar to B, gene ontology terms for cellular component.

S5

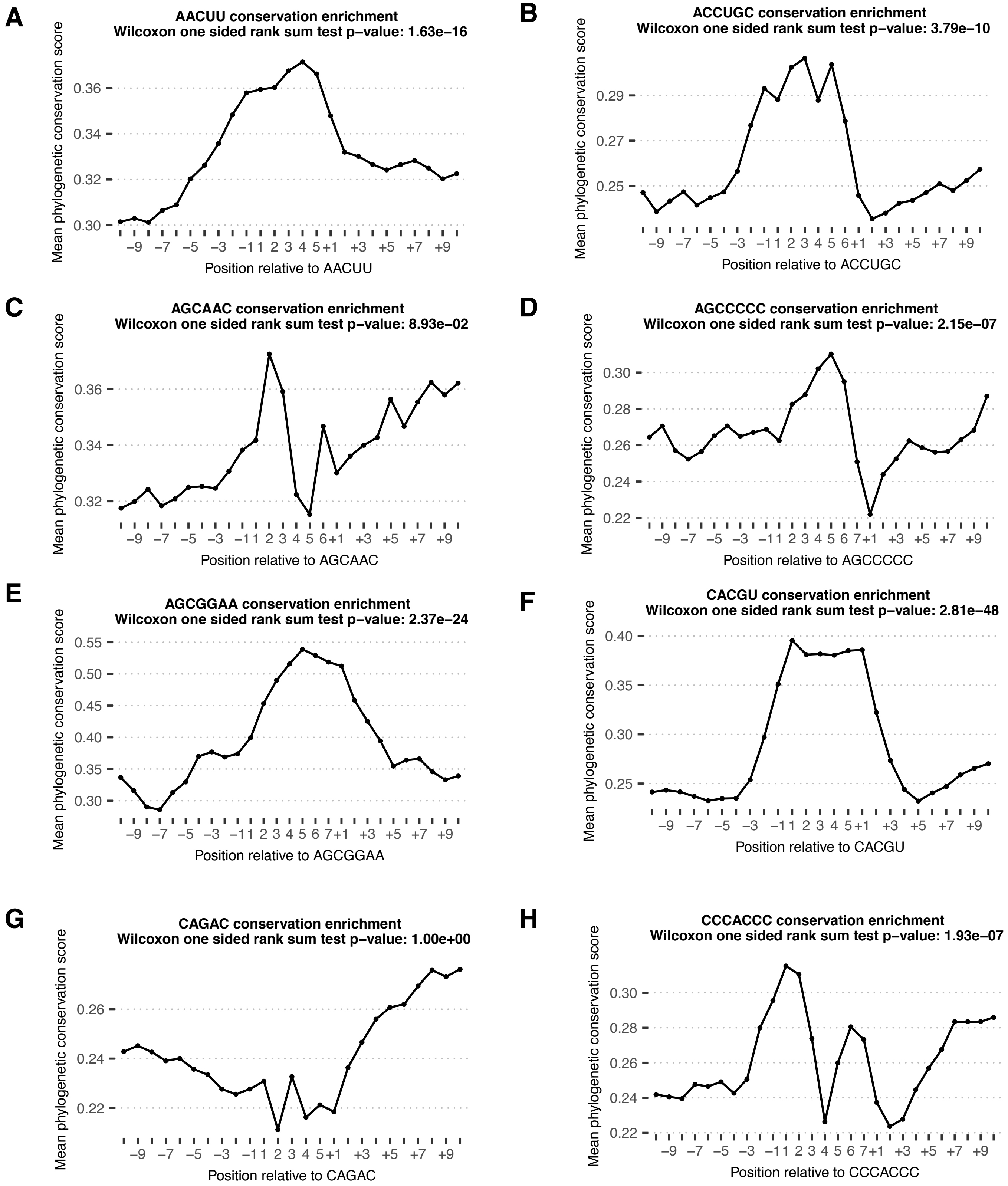
A
AACUU conservation enrichment
Wilcoxon one sided rank sum test p−value: 1.63e−16
Mean phylogenetic conservation score
Position relative to AACUU
B
ACCUGC conservation enrichment
Wilcoxon one sided rank sum test p−value: 3.79e−10
Mean phylogenetic conservation score
Position relative to ACCUGC
C
AGCAAC conservation enrichment
Wilcoxon one sided rank sum test p−value: 8.93e−02
Mean phylogenetic conservation score
Position relative to AGCAAC
D
AGCCCCC conservation enrichment
Wilcoxon one sided rank sum test p−value: 2.15e−07
Mean phylogenetic conservation score
Position relative to AGCCCCC
E
AGCGGAA conservation enrichment
Wilcoxon one sided rank sum test p−value: 2.37e−24
Mean phylogenetic conservation score
Position relative to AGCGGAA
F
CACGU conservation enrichment
Wilcoxon one sided rank sum test p−value: 2.81e−48
Mean phylogenetic conservation score
Position relative to CACGU
G
CAGAC conservation enrichment
Wilcoxon one sided rank sum test p−value: 1.00e+00
Mean phylogenetic conservation score
Position relative to CAGAC
H
CCCACCC conservation enrichment
Wilcoxon one sided rank sum test p−value: 1.93e−07
Mean phylogenetic conservation score
Position relative to CCCACCC

I

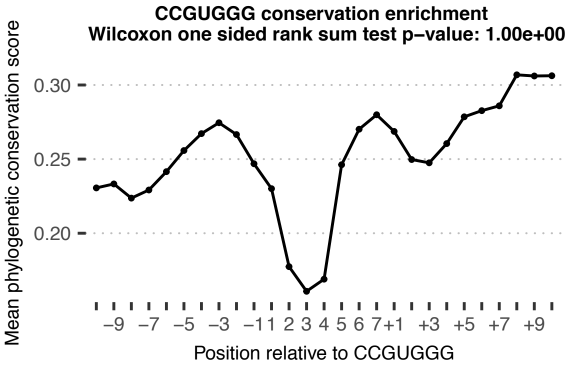
CCGUGGG conservation enrichment
Wilcoxon one sided rank sum test p−value: 1.00e+00
Mean phylogenetic conservation score
0.30
0.25
0.20
−9 −7 −5 −3 −1 1 2 3 4 5 6 7 +1 +3 +5 +7 +9
Position relative to CCGUGGG

J

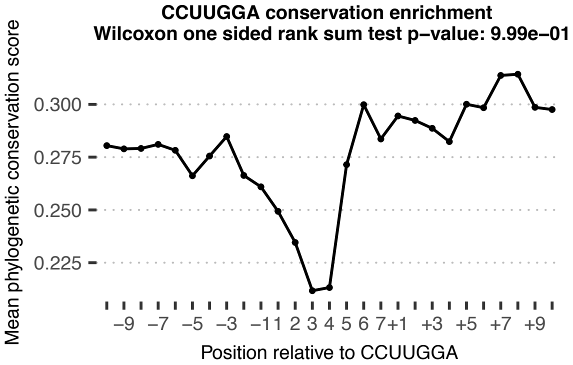
CCUUGGA conservation enrichment
Wilcoxon one sided rank sum test p−value: 9.99e−01
Mean phylogenetic conservation score
0.300
0.275
0.250
0.225
−9 −7 −5 −3 −1 1 2 3 4 5 6 7 +1 +3 +5 +7 +9
Position relative to CCUUGGA

K

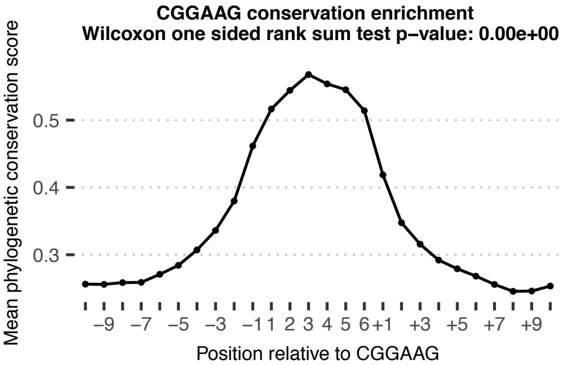
CGGAAG conservation enrichment
Wilcoxon one sided rank sum test p−value: 0.00e+00
Mean phylogenetic conservation score
0.5
0.4
0.3
−9 −7 −5 −3 −1 1 2 3 4 5 6 +1 +3 +5 +7 +9
Position relative to CGGAAG

L

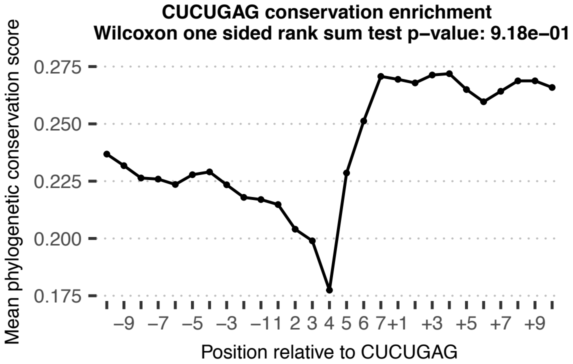
CUCUGAG conservation enrichment
Wilcoxon one sided rank sum test p−value: 9.18e−01
Mean phylogenetic conservation score
0.275
0.250
0.225
0.200
0.175
−9 −7 −5 −3 −1 1 2 3 4 5 6 7 +1 +3 +5 +7 +9
Position relative to CUCUGAG

M

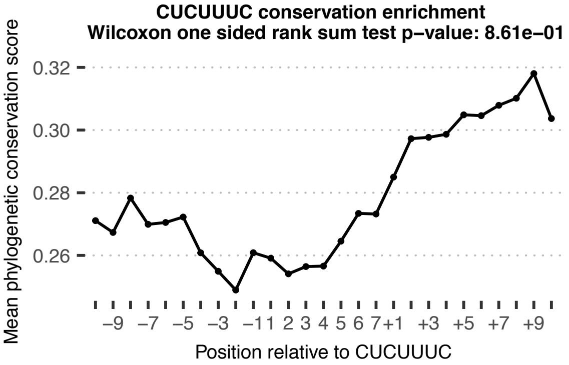
CUCUUUC conservation enrichment
Wilcoxon one sided rank sum test p−value: 8.61e−01
Mean phylogenetic conservation score
0.32
0.30
0.28
0.26
−9 −7 −5 −3 −1 1 2 3 4 5 6 7 +1 +3 +5 +7 +9
Position relative to CUCUUUC

N

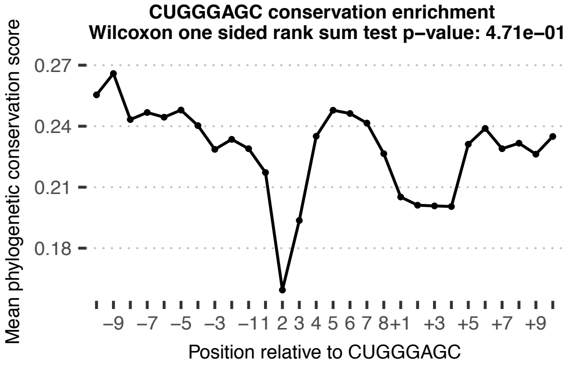
CUGGGAGC conservation enrichment
Wilcoxon one sided rank sum test p−value: 4.71e−01
Mean phylogenetic conservation score
0.27
0.24
0.21
0.18
−9 −7 −5 −3 −1 1 2 3 4 5 6 7 8 +1 +3 +5 +7 +9
Position relative to CUGGGAGC

O

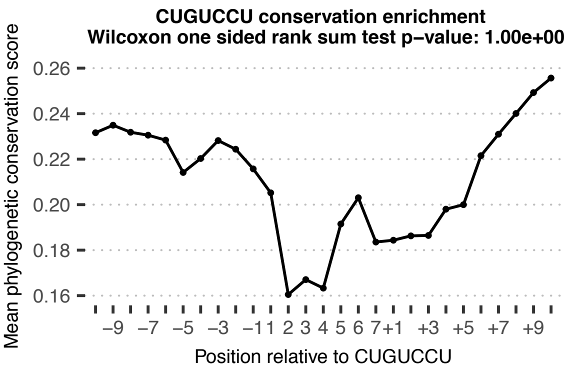
CUGUCCU conservation enrichment
Wilcoxon one sided rank sum test p−value: 1.00e+00
Mean phylogenetic conservation score
0.26
0.24
0.22
0.20
0.18
0.16
−9 −7 −5 −3 −1 1 2 3 4 5 6 7 +1 +3 +5 +7 +9
Position relative to CUGUCCU

P

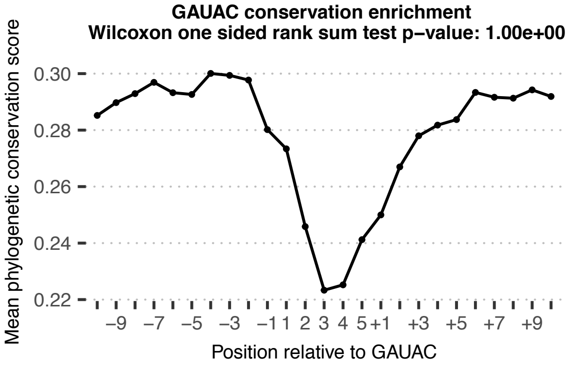
GAUAC conservation enrichment
Wilcoxon one sided rank sum test p−value: 1.00e+00
Mean phylogenetic conservation score
0.30
0.28
0.26
0.24
0.22
−9 −7 −5 −3 −1 1 2 3 4 5 +1 +3 +5 +7 +9
Position relative to GAUAC

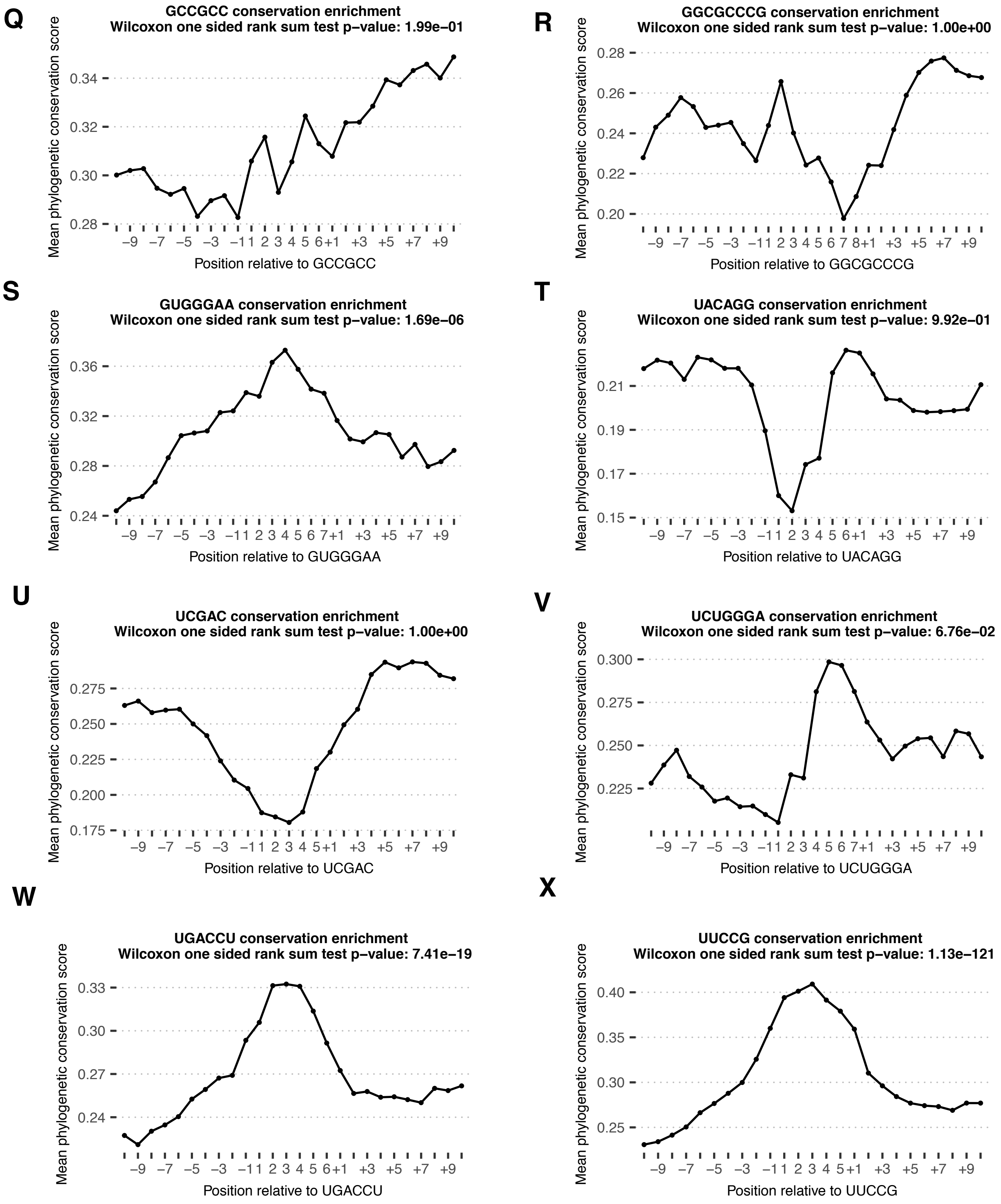

Figure S5: Average 100-vertebrate PhastCons score (y-axis, Materials and Methods) per position relative to the exact motif match instances in 5' UTR (x-axis). P-values assess significance of the average 100-vertebrate PhastCons scores at the motif sites compared to the two 10-nucleotide flanking regions (Materials and Methods).

S6

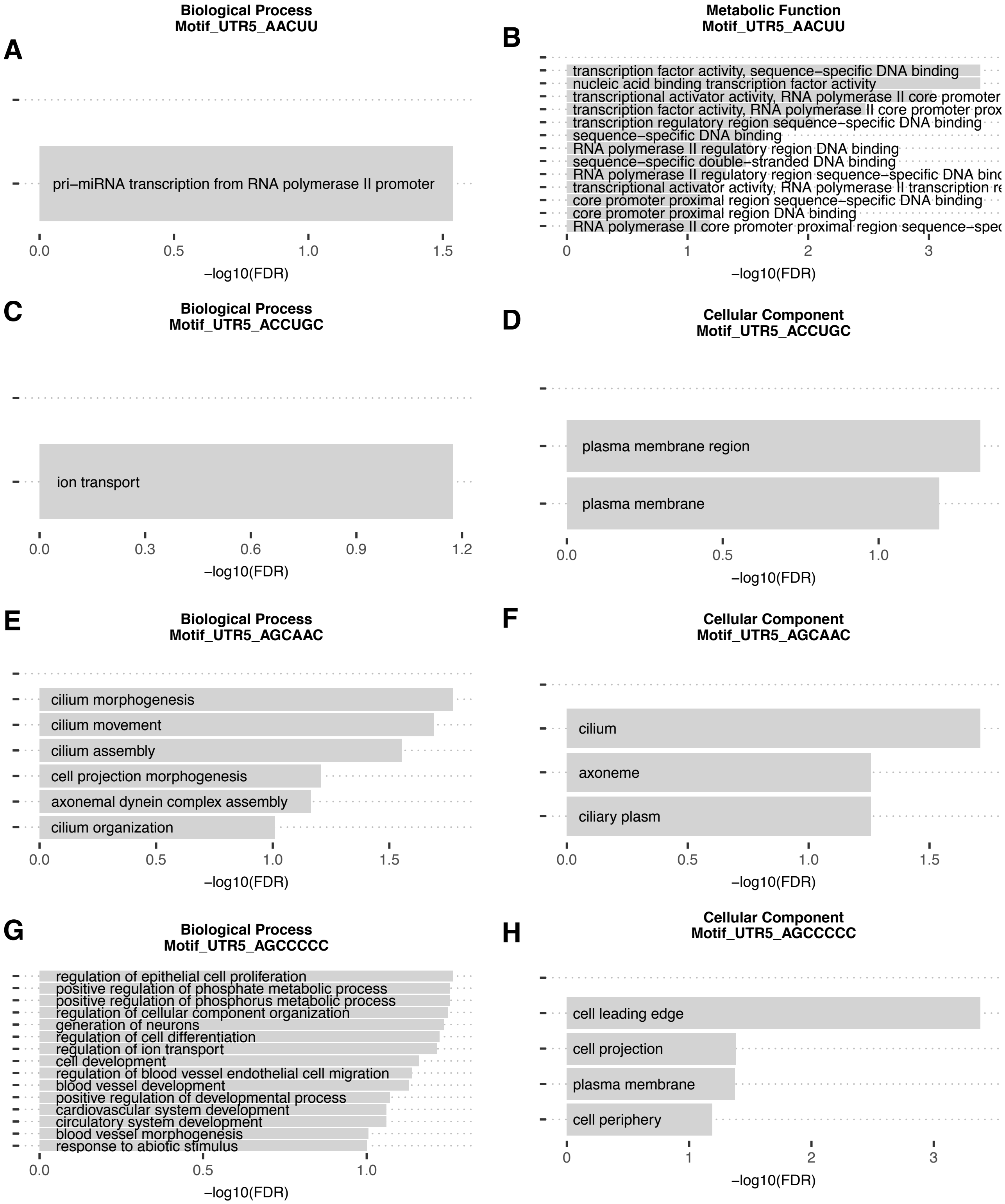

A
Biological Process
Motif_UTR5_AACUU
pri−miRNA transcription from RNA polymerase II promoter
0.0
0.5
1.0
1.5
−log10(FDR)
B
Metabolic Function
Motif_UTR5_AACUU
transcription factor activity, sequence−specific DNA binding
nucleic acid binding transcription factor activity
transcriptional activator activity, RNA polymerase II core promoter
transcription factor activity, RNA polymerase II core promoter prox
transcription regulatory region sequence−specific DNA binding
sequence−specific DNA binding
RNA polymerase II regulatory region DNA binding
sequence−specific double−stranded DNA binding
RNA polymerase II regulatory region sequence−specific DNA binc
transcriptional activator activity, RNA polymerase II transcription re
core promoter proximal region sequence−specific DNA binding
core promoter proximal region DNA binding
RNA polymerase II core promoter proximal region sequence−spec
0
1
2
3
−log10(FDR)
C
Biological Process
Motif_UTR5_ACCUGC
ion transport
0.0
0.3
0.6
0.9
1.2
−log10(FDR)
D
Cellular Component
Motif_UTR5_ACCUGC
plasma membrane region
plasma membrane
0.0
0.5
1.0
−log10(FDR)
E
Biological Process
Motif_UTR5_AGCAAC
cilium morphogenesis
cilium movement
cilium assembly
cell projection morphogenesis
axonemal dynein complex assembly
cilium organization
0.0
0.5
1.0
1.5
−log10(FDR)
F
Cellular Component
Motif_UTR5_AGCAAC
cilium
axoneme
ciliary plasm
0.0
0.5
1.0
1.5
−log10(FDR)
G
Biological Process
Motif_UTR5_AGCCCCC
regulation of epithelial cell proliferation
positive regulation of phosphate metabolic process
positive regulation of phosphorus metabolic process
regulation of cellular component organization
generation of neurons
regulation of cell differentiation
regulation of ion transport
cell development
regulation of blood vessel endothelial cell migration
blood vessel development
positive regulation of developmental process
cardiovascular system development
circulatory system development
blood vessel morphogenesis
response to abiotic stimulus
0.0
0.5
1.0
−log10(FDR)
H
Cellular Component
Motif_UTR5_AGCCCCC
cell leading edge
cell projection
plasma membrane
cell periphery
0
1
2
3
−log10(FDR)

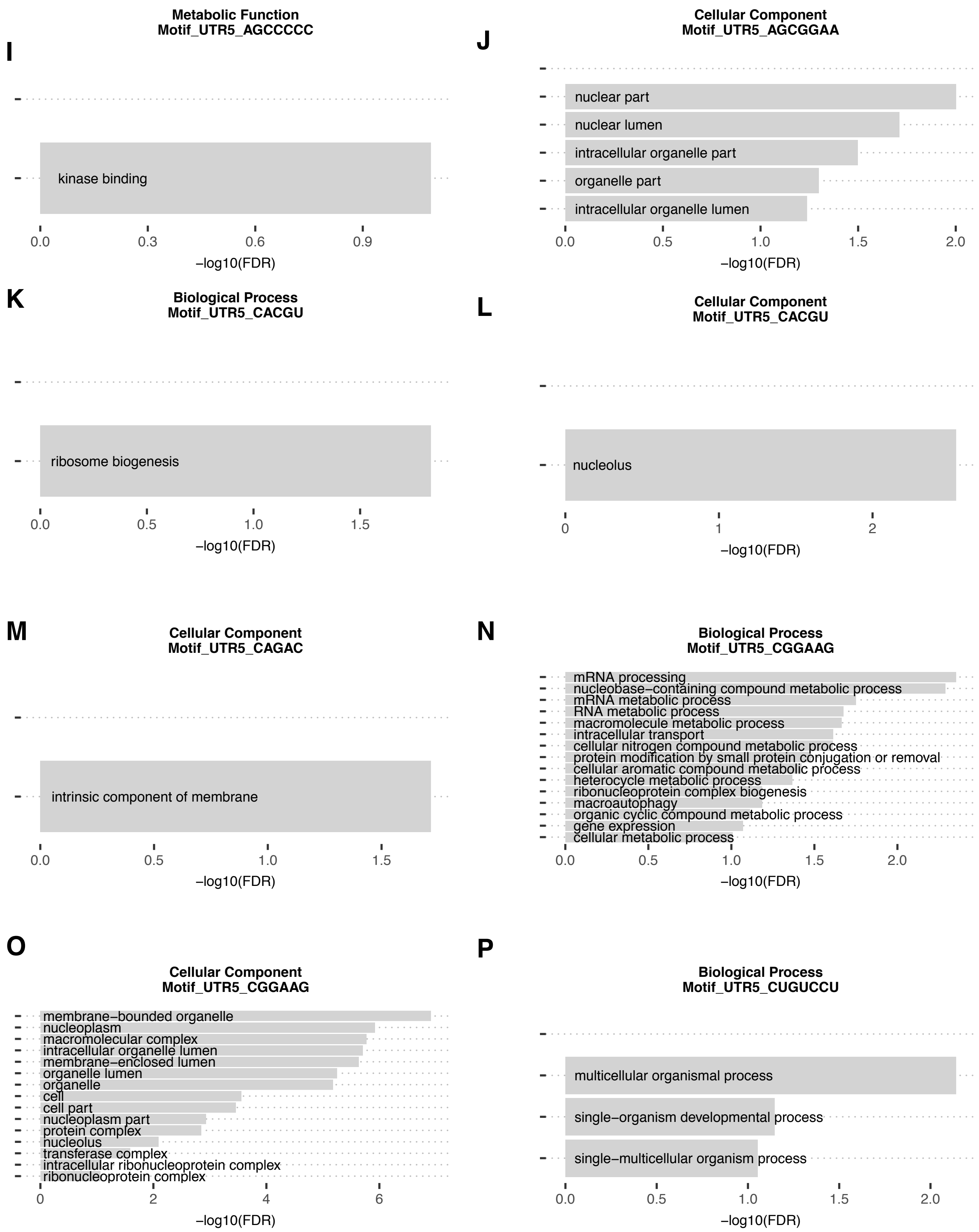
I
Metabolic Function
Motif_UTR5_AGCCCCC
kinase binding
0.0
0.3
0.6
0.9
−log10(FDR)
J
Cellular Component
Motif_UTR5_AGCGGAA
nuclear part
nuclear lumen
intracellular organelle part
organelle part
intracellular organelle lumen
0.0
0.5
1.0
1.5
2.0
−log10(FDR)
K
Biological Process
Motif_UTR5_CACGU
ribosome biogenesis
0.0
0.5
1.0
1.5
−log10(FDR)
L
Cellular Component
Motif_UTR5_CACGU
nucleolus
0
1
2
−log10(FDR)
M
Cellular Component
Motif_UTR5_CAGAC
intrinsic component of membrane
0.0
0.5
1.0
1.5
−log10(FDR)
N
Biological Process
Motif_UTR5_CGGAAG
mRNA processing
nucleobase−containing compound metabolic process
mRNA metabolic process
RNA metabolic process
macromolecule metabolic process
intracellular transport
cellular nitrogen compound metabolic process
protein modification by small protein conjugation or removal
cellular aromatic compound metabolic process
heterocycle metabolic process
ribonucleoprotein complex biogenesis
macroautophagy
organic cyclic compound metabolic process
gene expression
cellular metabolic process
0.0
0.5
1.0
1.5
2.0
−log10(FDR)
O
Cellular Component
Motif_UTR5_CGGAAG
membrane−bounded organelle
nucleoplasm
macromolecular complex
intracellular organelle lumen
membrane−enclosed lumen
organelle lumen
organelle
cell
cell part
nucleoplasm part
protein complex
nucleolus
transferase complex
intracellular ribonucleoprotein complex
ribonucleoprotein complex
0
2
4
6
−log10(FDR)
P
Biological Process
Motif_UTR5_CUGUCCU
multicellular organismal process
single−organism developmental process
single−multicellular organism process
0.0
0.5
1.0
1.5
2.0
−log10(FDR)

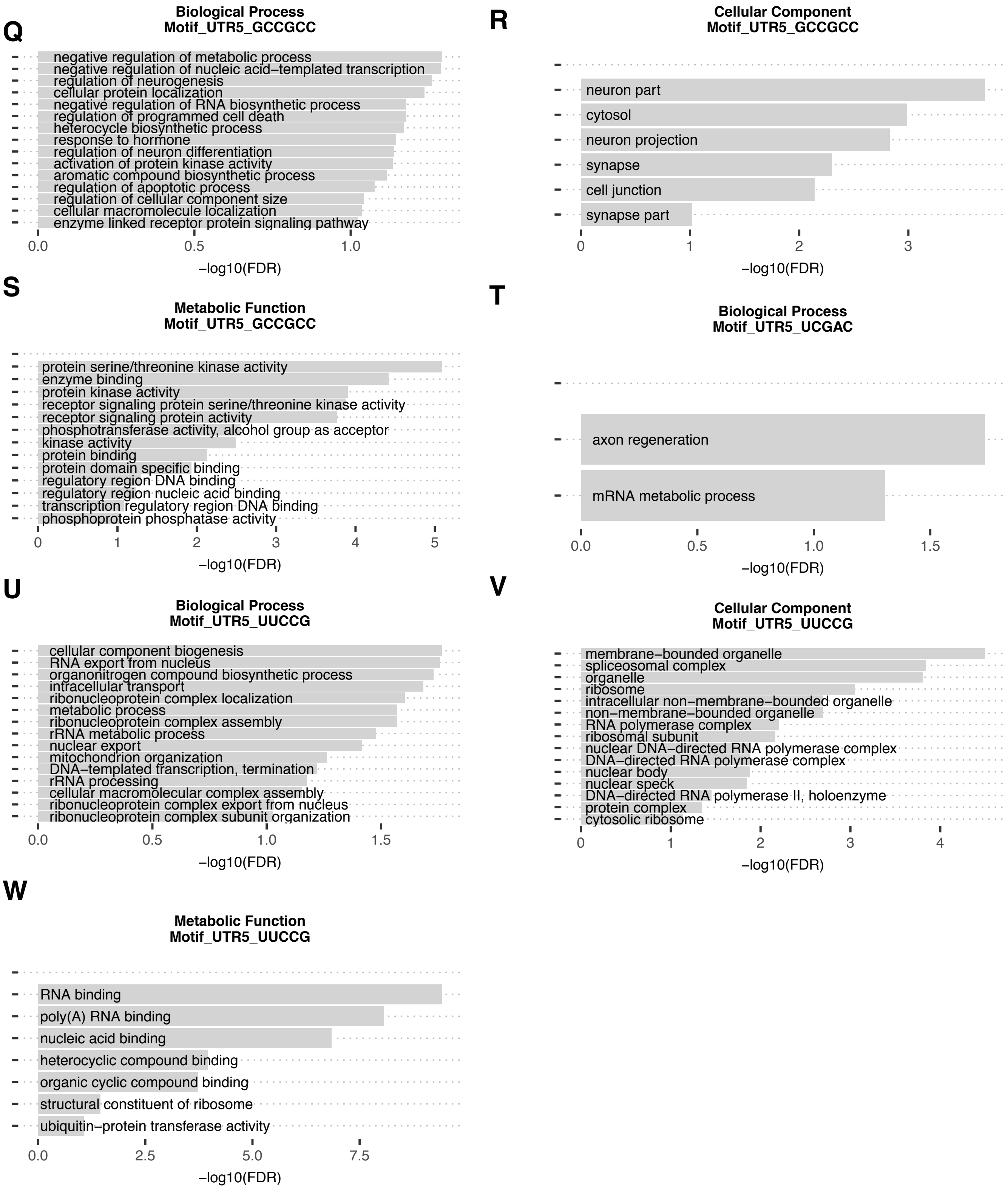

Figure S6: Gene ontology terms that are enriched for set of genes that contain consensus sequences of the de-novo identified k-mers in 5' UTR that are predictive of PTR ratios.

S7

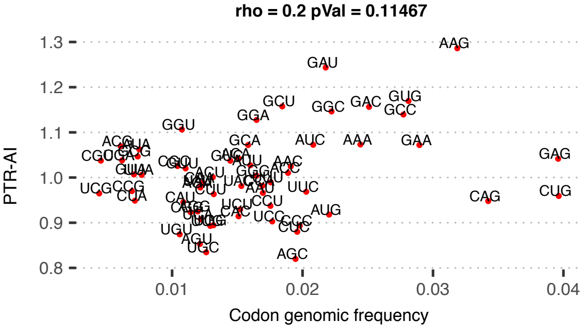

Figure S7: PTR-AI (2 fold codon frequency increase effect on PTR ratio) does not significantly correlate with human genomic codon frequencies.

S8

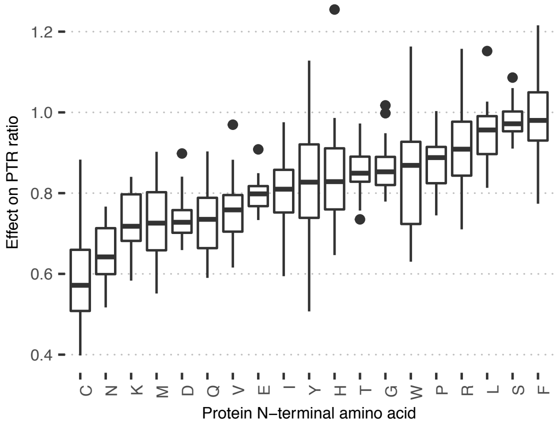

Figure S8: Distribution of the effects of protein N-terminal residues (with respect to Alanine) on PTR ratio across tissues.

S9

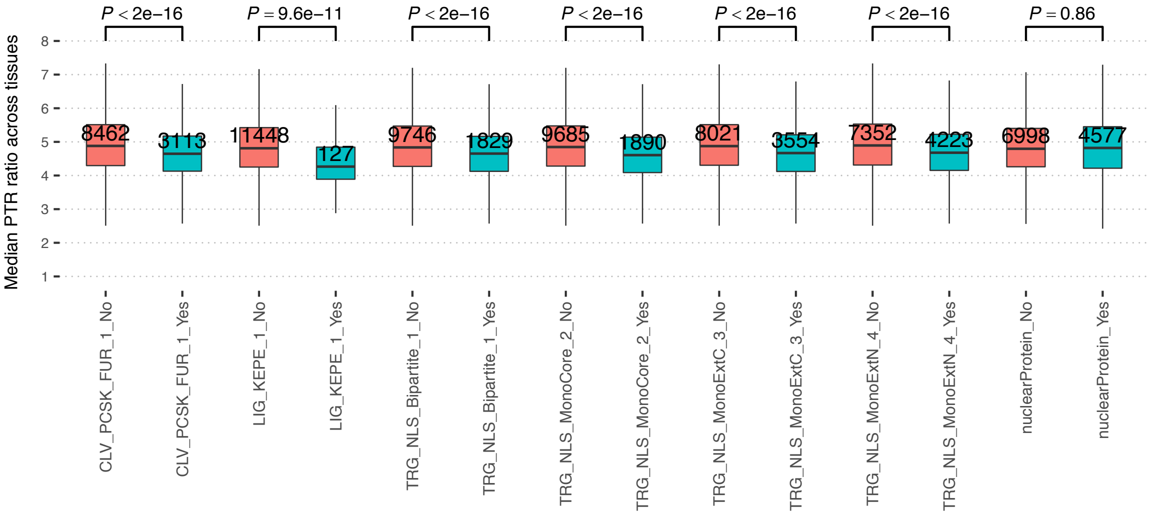

Figure S9: Distribution of median PTR ratio across 29 tissues for genes with and without the eukaryotic linear protein motifs. CLV_PKCS_FUR_1 (Furin (PACE) cleavage site), LIG_KEPE_1 (Sumoylation site), TRG_NLS_BIPARTITE_1 (classical bipartite nuclear localization signal), three classical monopartite nuclear localisation signals: TRG_NLS_MonoCore_2, TRG_NLS_MonoExtC_3 , TRG_NLS_MonoExtN_4, and nuclear proteins (GO:0005634) in general. Four nuclear localization signals were associated with less median PTR ratio even though there is no significant PTR ratio difference between nuclear and non-nuclear proteins.

S10

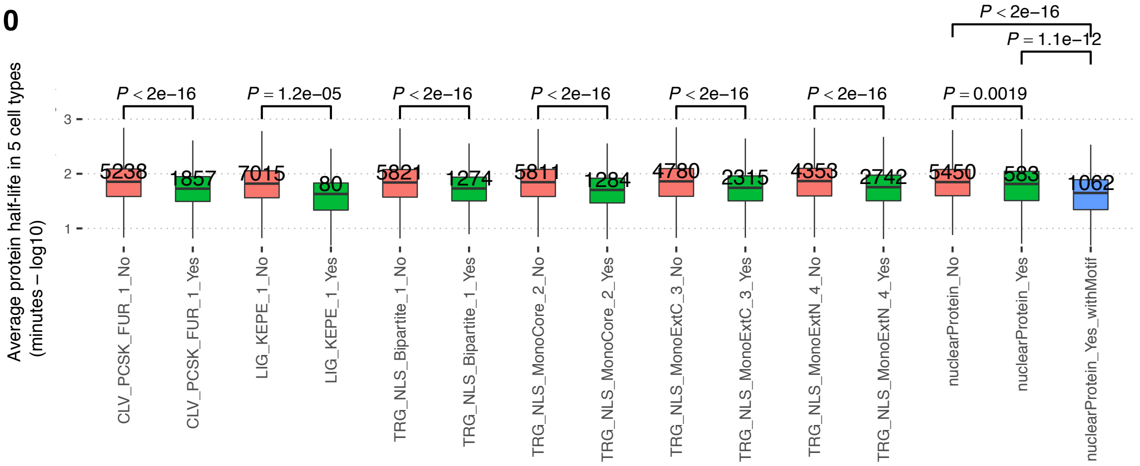

Figure S10: Same as S9, for average protein half-lives across HeLa cells, B cells, NK Cells, Hepatocytes and Monocytes. Nuclear proteins with the four nuclear localization signals we have identified have even shorter protein half-lives compared to other nuclear proteins.

S11

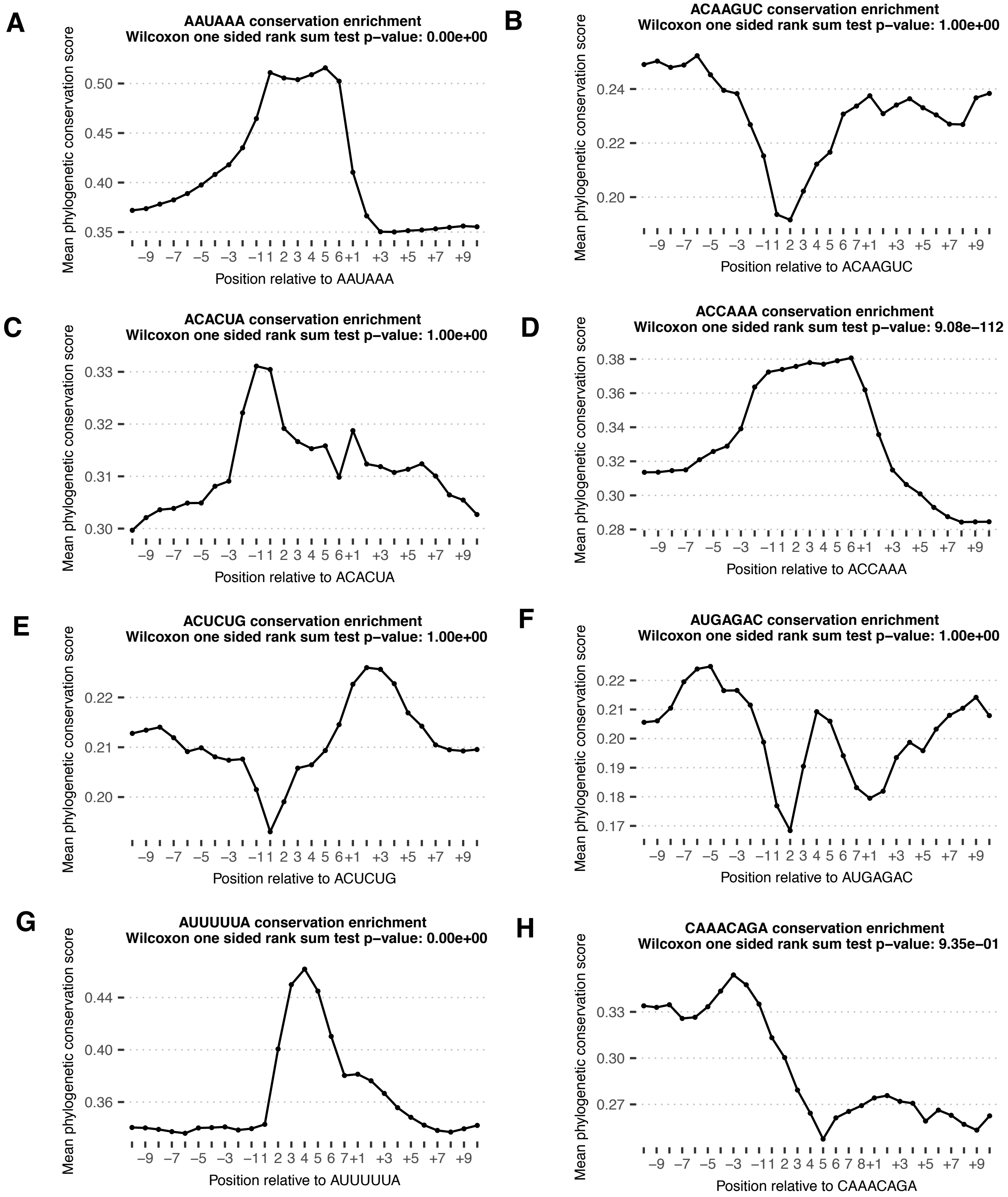
A
AAUAAA conservation enrichment
Wilcoxon one sided rank sum test p−value: 0.00e+00
Mean phylogenetic conservation score
0.35
0.40
0.45
0.50
−9 −7 −5 −3 −11 2 3 4 5 6+1 +3 +5 +7 +9
Position relative to AAUAAA
B
ACAAGUC conservation enrichment
Wilcoxon one sided rank sum test p−value: 1.00e+00
Mean phylogenetic conservation score
0.20
0.22
0.24
−9 −7 −5 −3 −11 2 3 4 5 6 7+1 +3 +5 +7 +9
Position relative to ACAAGUC
C
ACACUA conservation enrichment
Wilcoxon one sided rank sum test p−value: 1.00e+00
Mean phylogenetic conservation score
0.30
0.31
0.32
0.33
−9 −7 −5 −3 −11 2 3 4 5 6+1 +3 +5 +7 +9
Position relative to ACACUA
D
ACCAAA conservation enrichment
Wilcoxon one sided rank sum test p−value: 9.08e−112
Mean phylogenetic conservation score
0.28
0.30
0.32
0.34
0.36
0.38
−9 −7 −5 −3 −11 2 3 4 5 6+1 +3 +5 +7 +9
Position relative to ACCAAA
E
ACUCUG conservation enrichment
Wilcoxon one sided rank sum test p−value: 1.00e+00
Mean phylogenetic conservation score
0.20
0.21
0.22
−9 −7 −5 −3 −11 2 3 4 5 6+1 +3 +5 +7 +9
Position relative to ACUCUG
F
AUGAGAC conservation enrichment
Wilcoxon one sided rank sum test p−value: 1.00e+00
Mean phylogenetic conservation score
0.17
0.18
0.19
0.20
0.21
0.22
−9 −7 −5 −3 −11 2 3 4 5 6 7+1 +3 +5 +7 +9
Position relative to AUGAGAC
G
AUUUUUA conservation enrichment
Wilcoxon one sided rank sum test p−value: 0.00e+00
Mean phylogenetic conservation score
0.36
0.40
0.44
−9 −7 −5 −3 −11 2 3 4 5 6 7+1 +3 +5 +7 +9
Position relative to AUUUUUA
H
CAAACAGA conservation enrichment
Wilcoxon one sided rank sum test p−value: 9.35e−01
Mean phylogenetic conservation score
0.27
0.30
0.33
−9 −7 −5 −3 −11 2 3 4 5 6 7 8+1 +3 +5 +7 +9
Position relative to CAAACAGA

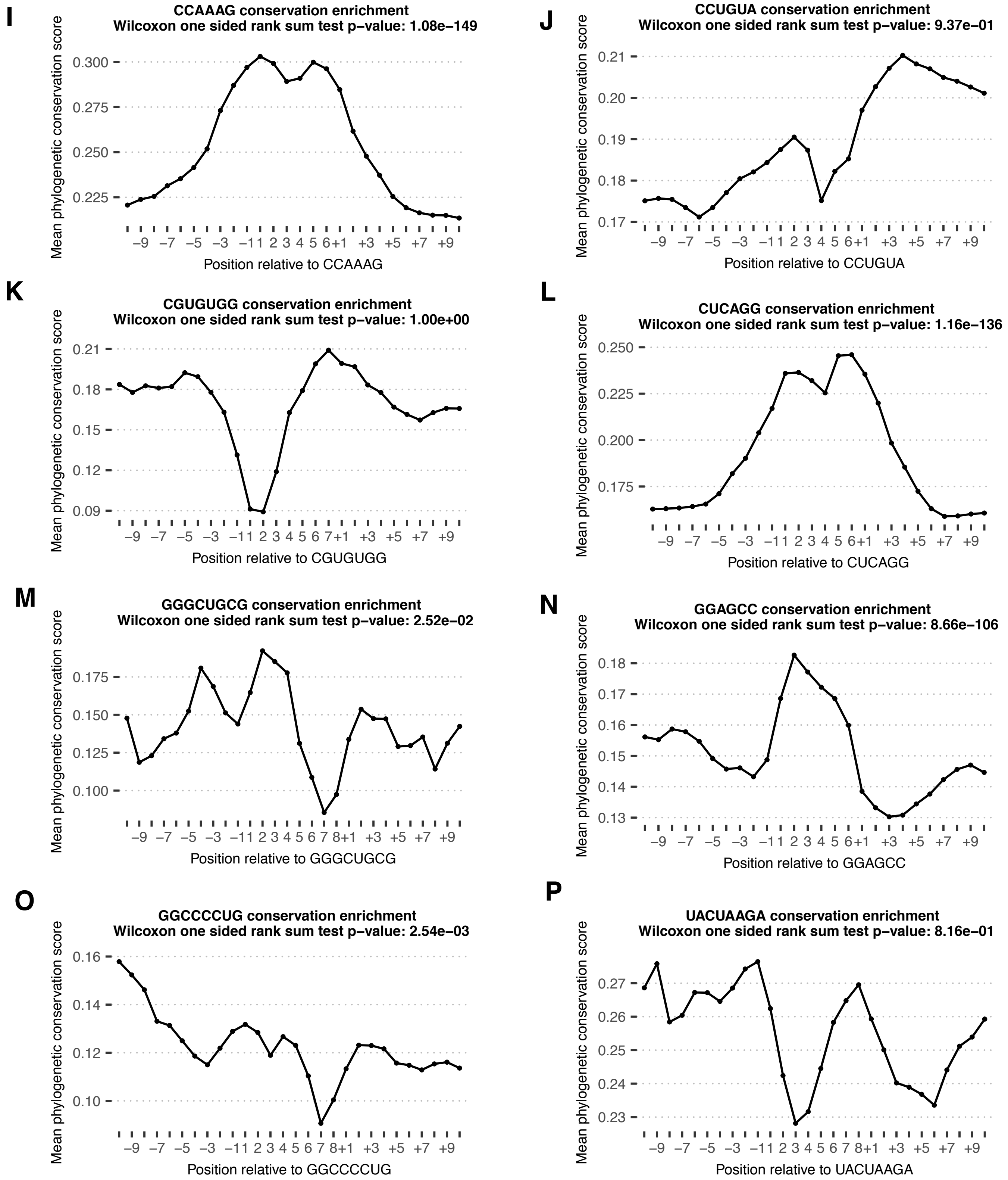
I
CCAAAG conservation enrichment
Wilcoxon one sided rank sum test p−value: 1.08e−149
Mean phylogenetic conservation score
0.300
0.275
0.250
0.225
−9 −7 −5 −3 −1 1 2 3 4 5 6 +1 +3 +5 +7 +9
Position relative to CCAAAG
J
CCUGUA conservation enrichment
Wilcoxon one sided rank sum test p−value: 9.37e−01
Mean phylogenetic conservation score
0.21
0.20
0.19
0.18
0.17
−9 −7 −5 −3 −1 1 2 3 4 5 6 +1 +3 +5 +7 +9
Position relative to CCUGUA
K
CGUGUGG conservation enrichment
Wilcoxon one sided rank sum test p−value: 1.00e+00
Mean phylogenetic conservation score
0.21
0.18
0.15
0.12
0.09
−9 −7 −5 −3 −1 1 2 3 4 5 6 7 +1 +3 +5 +7 +9
Position relative to CGUGUGG
L
CUCAGG conservation enrichment
Wilcoxon one sided rank sum test p−value: 1.16e−136
Mean phylogenetic conservation score
0.250
0.225
0.200
0.175
−9 −7 −5 −3 −1 1 2 3 4 5 6 +1 +3 +5 +7 +9
Position relative to CUCAGG
M
GGGCUGCG conservation enrichment
Wilcoxon one sided rank sum test p−value: 2.52e−02
Mean phylogenetic conservation score
0.175
0.150
0.125
0.100
−9 −7 −5 −3 −1 1 2 3 4 5 6 7 8 +1 +3 +5 +7 +9
Position relative to GGGCUGCG
N
GGAGCC conservation enrichment
Wilcoxon one sided rank sum test p−value: 8.66e−106
Mean phylogenetic conservation score
0.18
0.17
0.16
0.15
0.14
0.13
−9 −7 −5 −3 −1 1 2 3 4 5 6 +1 +3 +5 +7 +9
Position relative to GGAGCC
O
GGCCCCUG conservation enrichment
Wilcoxon one sided rank sum test p−value: 2.54e−03
Mean phylogenetic conservation score
0.16
0.14
0.12
0.10
−9 −7 −5 −3 −1 1 2 3 4 5 6 7 8 +1 +3 +5 +7 +9
Position relative to GGCCCCUG
P
UACUAAGA conservation enrichment
Wilcoxon one sided rank sum test p−value: 8.16e−01
Mean phylogenetic conservation score
0.27
0.26
0.25
0.24
0.23
−9 −7 −5 −3 −1 1 2 3 4 5 6 7 8 +1 +3 +5 +7 +9
Position relative to UACUAAGA

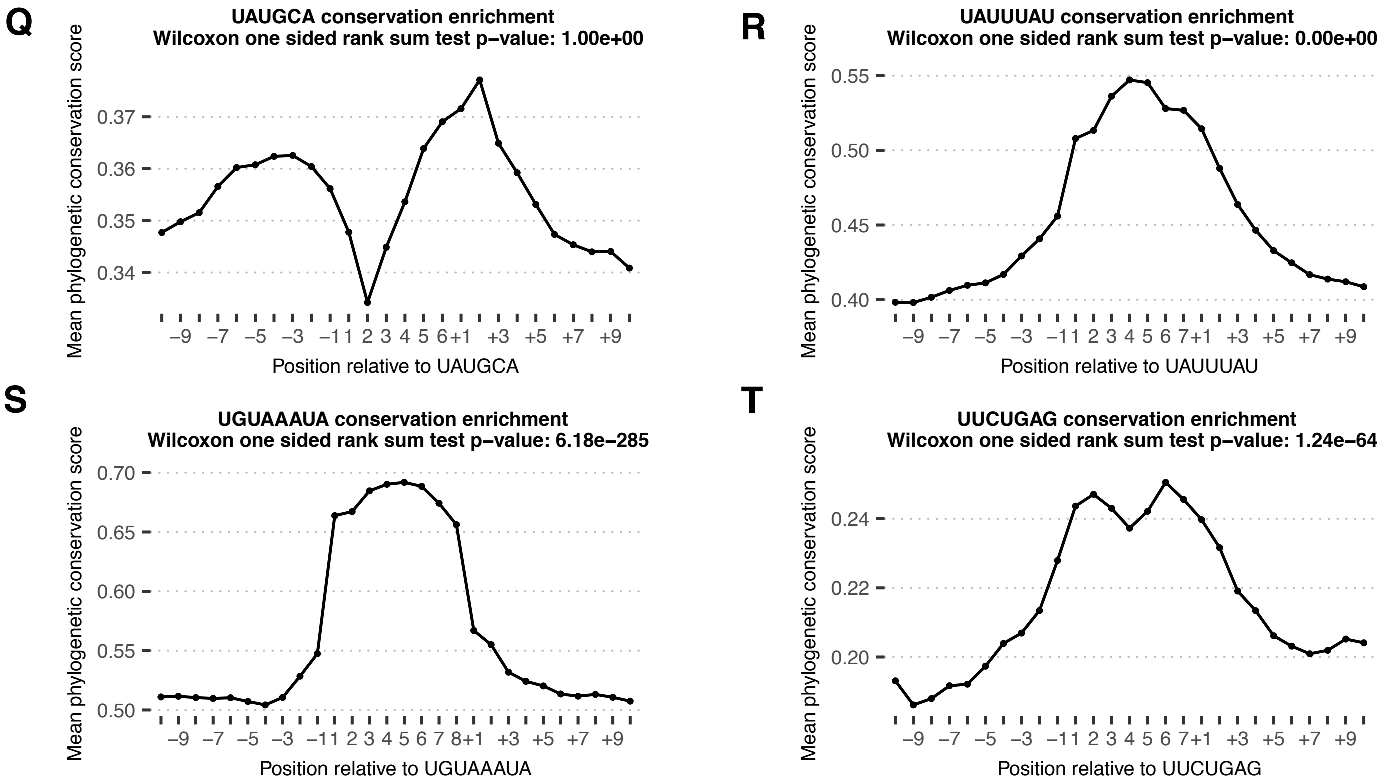

Figure S11: Average 100-vertebrate PhastCons score (y-axis, Materials and Methods) per position relative to the exact motif match instances in 3’ UTR (x-axis). P-values assess significance of the average 100-vertebrate PhastCons scores at the motif sites compared to the two 10-nucleotide flanking regions (Materials and Methods).

S12

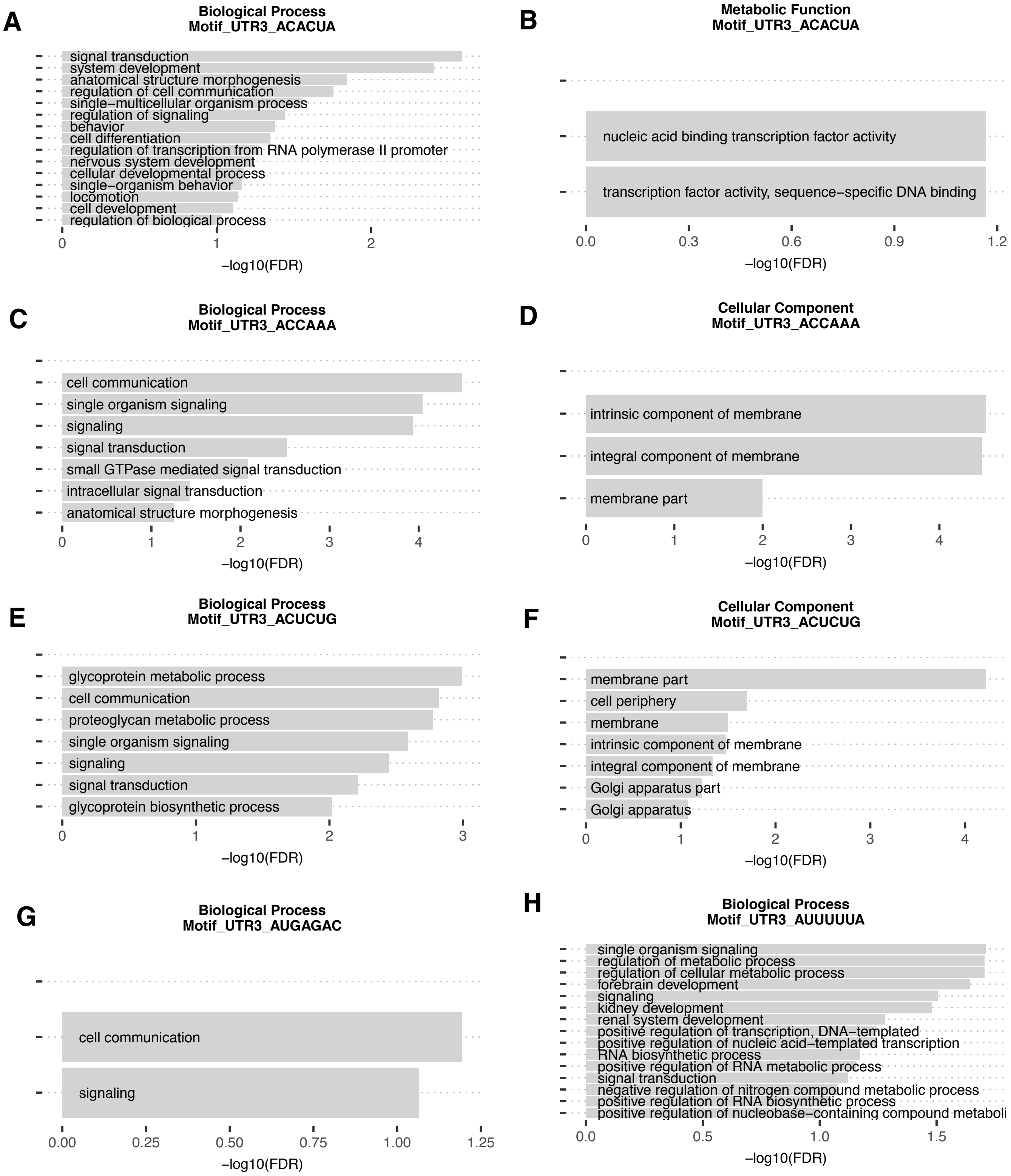
A
Biological Process
Motif_UTR3_ACACUA
signal transduction
system development
anatomical structure morphogenesis
regulation of cell communication
single−multicellular organism process
regulation of signaling
behavior
cell differentiation
regulation of transcription from RNA polymerase II promoter
nervous system development
cellular developmental process
single−organism behavior
locomotion
cell development
regulation of biological process
0
1
2
−log10(FDR)
B
Metabolic Function
Motif_UTR3_ACACUA
nucleic acid binding transcription factor activity
transcription factor activity, sequence−specific DNA binding
0.0
0.3
0.6
0.9
1.2
−log10(FDR)
C
Biological Process
Motif_UTR3_ACCAAA
cell communication
single organism signaling
signaling
signal transduction
small GTPase mediated signal transduction
intracellular signal transduction
anatomical structure morphogenesis
0
1
2
3
4
−log10(FDR)
D
Cellular Component
Motif_UTR3_ACCAAA
intrinsic component of membrane
integral component of membrane
membrane part
0
1
2
3
4
−log10(FDR)
E
Biological Process
Motif_UTR3_ACUCUG
glycoprotein metabolic process
cell communication
proteoglycan metabolic process
single organism signaling
signaling
signal transduction
glycoprotein biosynthetic process
0
1
2
3
−log10(FDR)
F
Cellular Component
Motif_UTR3_ACUCUG
membrane part
cell periphery
membrane
intrinsic component of membrane
integral component of membrane
Golgi apparatus part
Golgi apparatus
0
1
2
3
4
−log10(FDR)
G
Biological Process
Motif_UTR3_AUGAGAC
cell communication
signaling
0.00
0.25
0.50
0.75
1.00
1.25
−log10(FDR)
H
Biological Process
Motif_UTR3_AUUUUUA
single organism signaling
regulation of metabolic process
regulation of cellular metabolic process
forebrain development
signaling
kidney development
renal system development
positive regulation of transcription, DNA−templated
positive regulation of nucleic acid−templated transcription
RNA biosynthetic process
positive regulation of RNA metabolic process
signal transduction
negative regulation of nitrogen compound metabolic process
positive regulation of RNA biosynthetic process
positive regulation of nucleobase−containing compound metaboli
0.0
0.5
1.0
1.5
−log10(FDR)

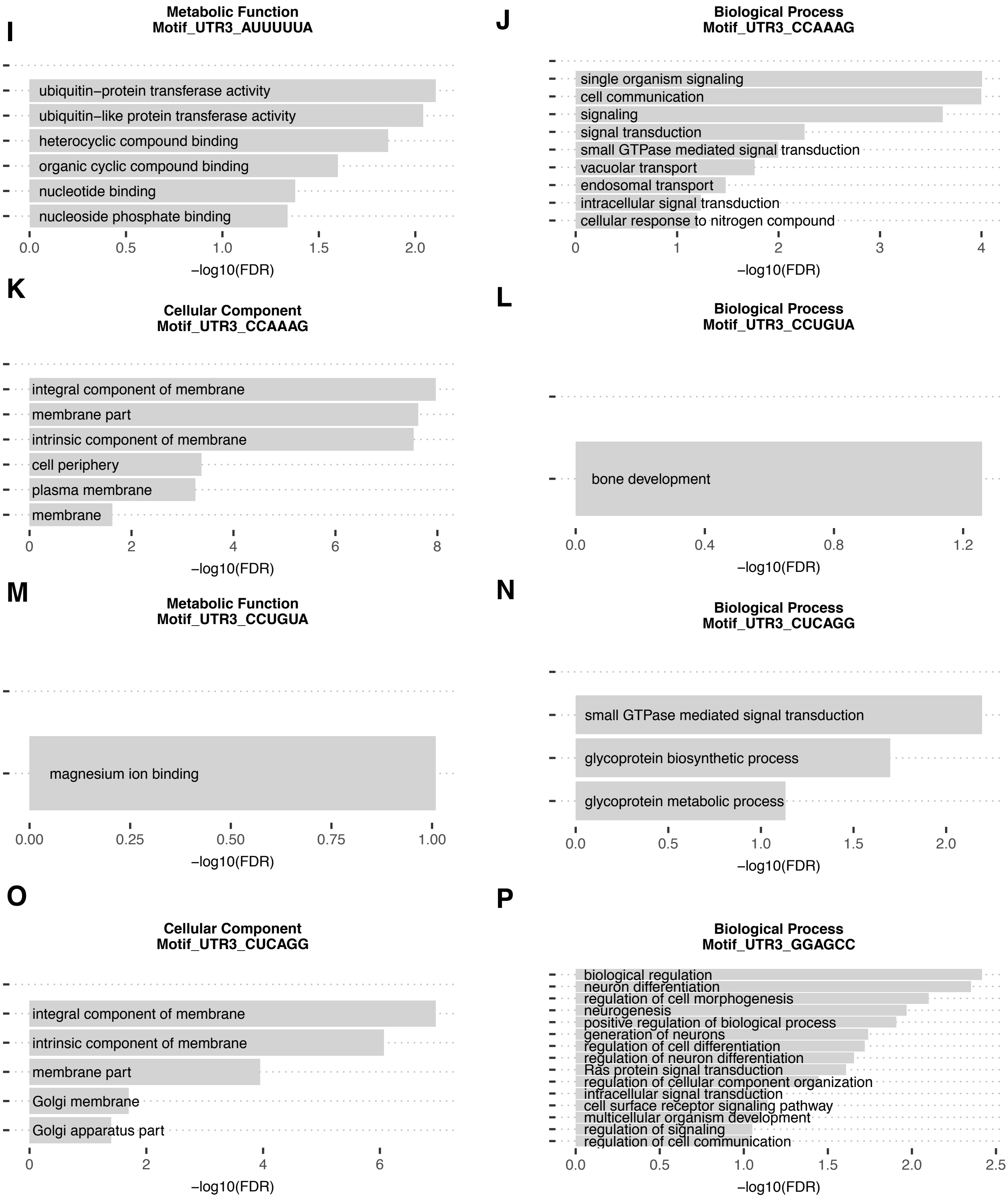
I
Metabolic Function
Motif_UTR3_AUUUUUA
ubiquitin−protein transferase activity
ubiquitin−like protein transferase activity
heterocyclic compound binding
organic cyclic compound binding
nucleotide binding
nucleoside phosphate binding
0.0
0.5
1.0
1.5
2.0
−log10(FDR)
J
Biological Process
Motif_UTR3_CCAAAG
single organism signaling
cell communication
signaling
signal transduction
small GTPase mediated signal transduction
vacuolar transport
endosomal transport
intracellular signal transduction
cellular response to nitrogen compound
0
1
2
3
4
−log10(FDR)
K
Cellular Component
Motif_UTR3_CCAAAG
integral component of membrane
membrane part
intrinsic component of membrane
cell periphery
plasma membrane
membrane
0
2
4
6
8
−log10(FDR)
L
Biological Process
Motif_UTR3_CCUGUA
bone development
0.0
0.4
0.8
1.2
−log10(FDR)
M
Metabolic Function
Motif_UTR3_CCUGUA
magnesium ion binding
0.00
0.25
0.50
0.75
1.00
−log10(FDR)
N
Biological Process
Motif_UTR3_CUCAGG
small GTPase mediated signal transduction
glycoprotein biosynthetic process
glycoprotein metabolic process
0.0
0.5
1.0
1.5
2.0
−log10(FDR)
O
Cellular Component
Motif_UTR3_CUCAGG
integral component of membrane
intrinsic component of membrane
membrane part
Golgi membrane
Golgi apparatus part
0
2
4
6
−log10(FDR)
P
Biological Process
Motif_UTR3_GGAGCC
biological regulation
neuron differentiation
regulation of cell morphogenesis
neurogenesis
positive regulation of biological process
generation of neurons
regulation of cell differentiation
regulation of neuron differentiation
Ras protein signal transduction
regulation of cellular component organization
intracellular signal transduction
cell surface receptor signaling pathway
multicellular organism development
regulation of signaling
regulation of cell communication
0.0
0.5
1.0
1.5
2.0
2.5
−log10(FDR)

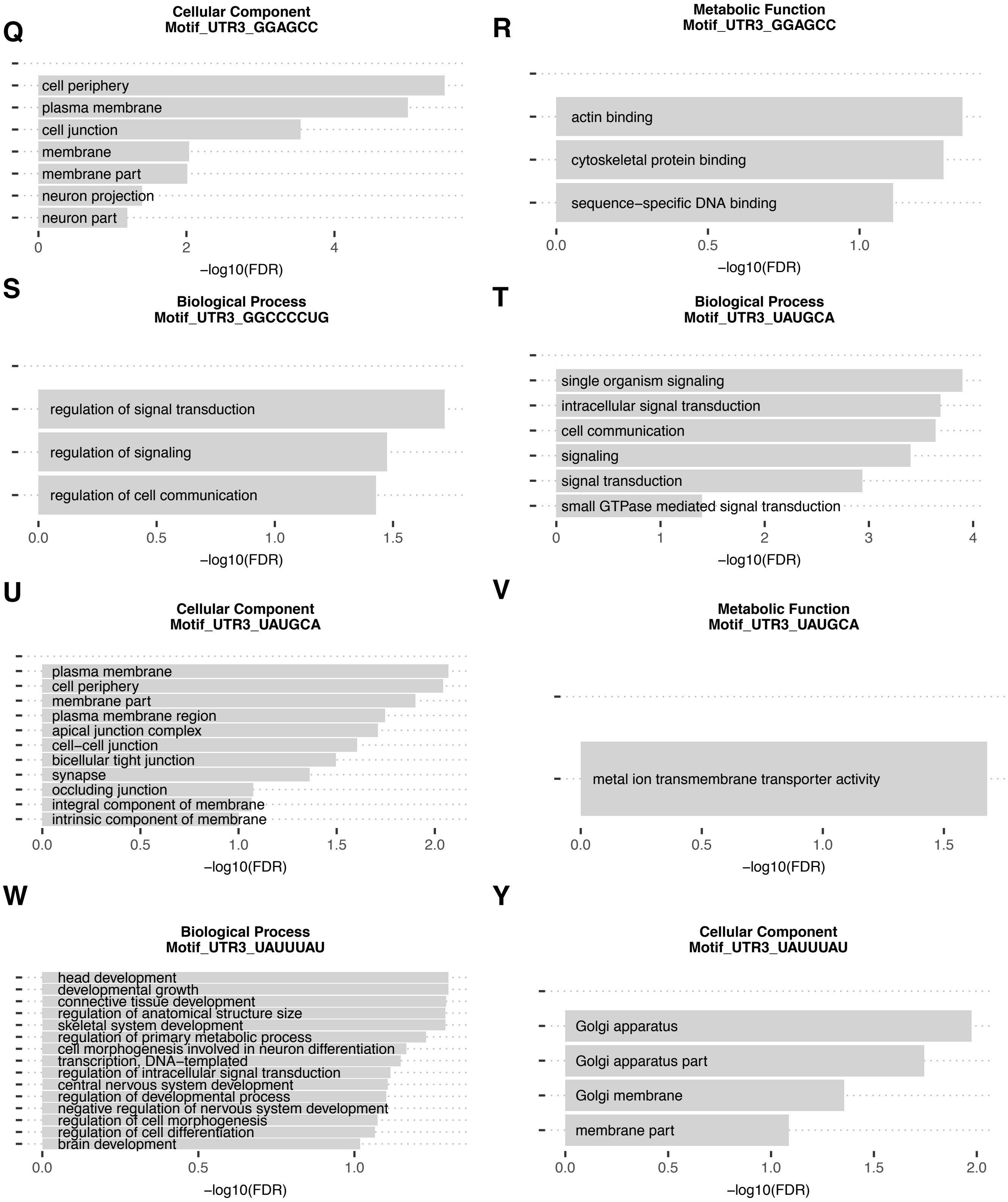
Q
Cellular Component
Motif_UTR3_GGAGCC
cell periphery
plasma membrane
cell junction
membrane
membrane part
neuron projection
neuron part
0
2
4
−log10(FDR)
R
Metabolic Function
Motif_UTR3_GGAGCC
actin binding
cytoskeletal protein binding
sequence−specific DNA binding
0.0
0.5
1.0
−log10(FDR)
S
Biological Process
Motif_UTR3_GGCCCCUG
regulation of signal transduction
regulation of signaling
regulation of cell communication
0.0
0.5
1.0
1.5
−log10(FDR)
T
Biological Process
Motif_UTR3_UAUGCA
single organism signaling
intracellular signal transduction
cell communication
signaling
signal transduction
small GTPase mediated signal transduction
0
1
2
3
4
−log10(FDR)
U
Cellular Component
Motif_UTR3_UAUGCA
plasma membrane
cell periphery
membrane part
plasma membrane region
apical junction complex
cell−cell junction
bicellular tight junction
synapse
occluding junction
integral component of membrane
intrinsic component of membrane
0.0
0.5
1.0
1.5
2.0
−log10(FDR)
V
Metabolic Function
Motif_UTR3_UAUGCA
metal ion transmembrane transporter activity
0.0
0.5
1.0
1.5
−log10(FDR)
W
Biological Process
Motif_UTR3_UAUUUAU
head development
developmental growth
connective tissue development
regulation of anatomical structure size
skeletal system development
regulation of primary metabolic process
cell morphogenesis involved in neuron differentiation
transcription, DNA−templated
regulation of intracellular signal transduction
central nervous system development
regulation of developmental process
negative regulation of nervous system development
regulation of cell morphogenesis
regulation of cell differentiation
brain development
0.0
0.5
1.0
−log10(FDR)
Y
Cellular Component
Motif_UTR3_UAUUUAU
Golgi apparatus
Golgi apparatus part
Golgi membrane
membrane part
0.0
0.5
1.0
1.5
2.0
−log10(FDR)

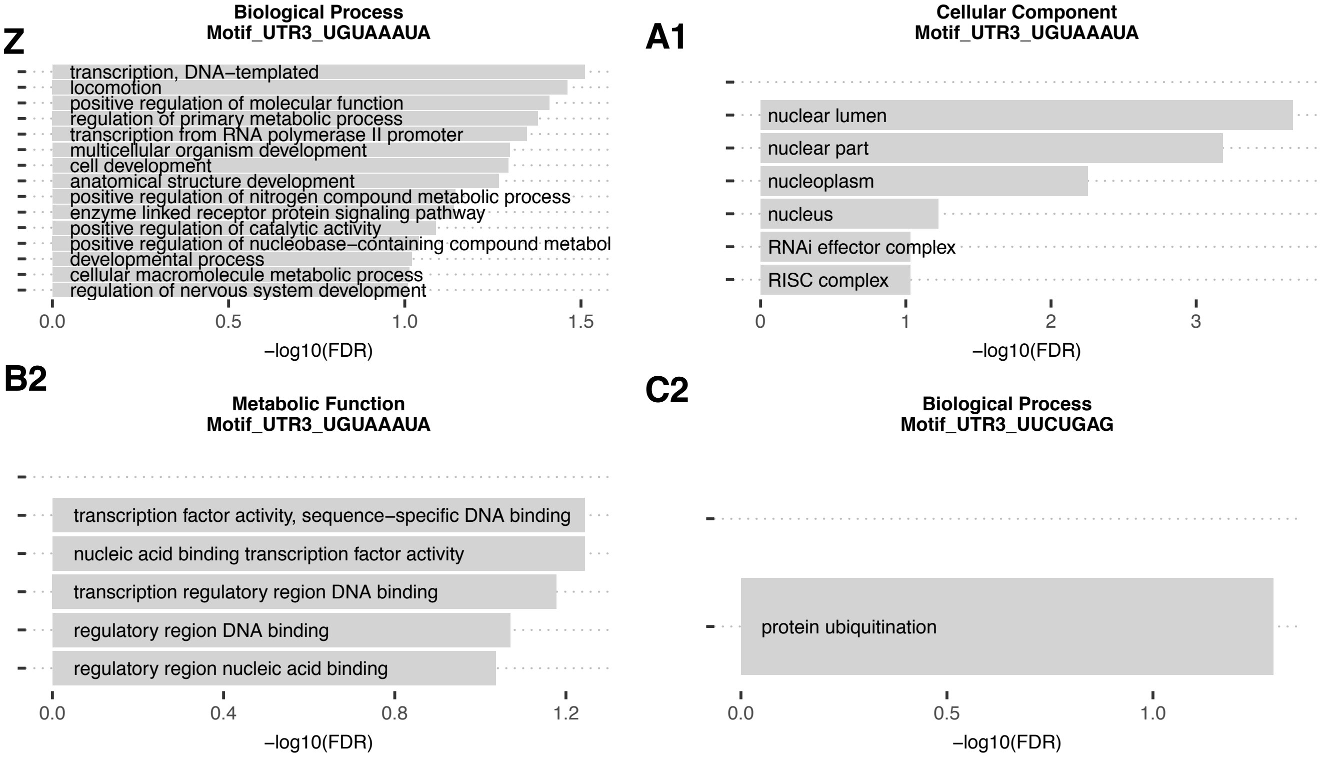

Figure S12: Gene ontology terms that are enriched for set of genes that contain consensus sequences of the de-novo identified k-mers in 3' UTR that are predictive of PTR ratios.

S13

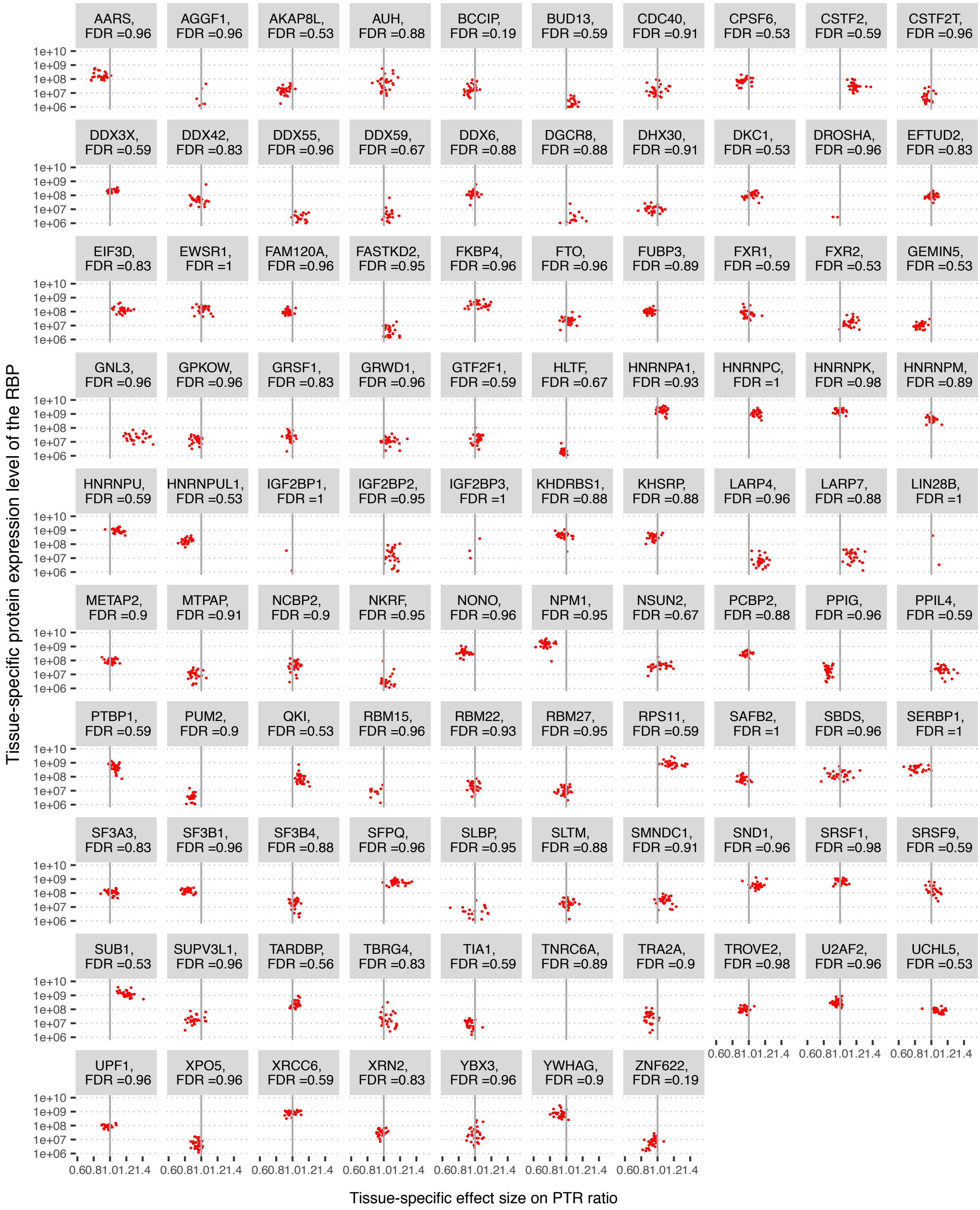

Figure S13: Our data set covers matched transcriptome and proteome measurements of 97 out of 112 RBPs whose target genes were detected by Van Nostrand et al. 81 of these RBPs were measured in all 29 tissues. The tissue-specific effect sizes of the RBP binding evidences in the linear model did not significantly correlate with the tissue-specific RBP expression levels.

S14

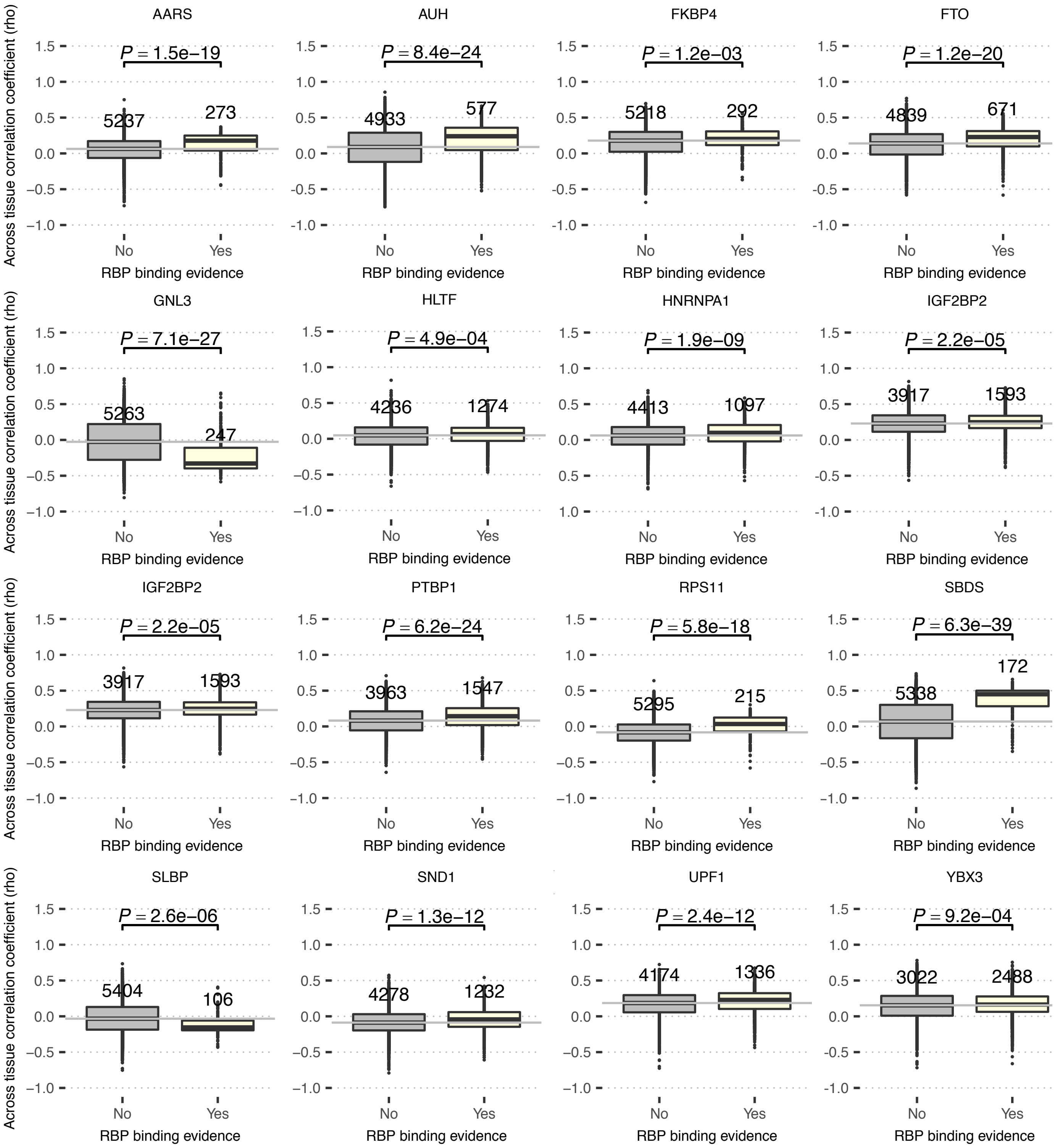

Figure S14: Distribution of Spearman's rho between RBP protein level expressions and target and non-target genes' PTR ratios across tissues. Only RBPs which are expressed in at least 15 tissues and with protein level standard deviation greater than 0.1 are taken into account. Similarly for the calculation of correlation coefficients, only genes with which are expressed in at least 15 tissues and with PTR ratio standard deviation greater than 0.1 are considered.

S15

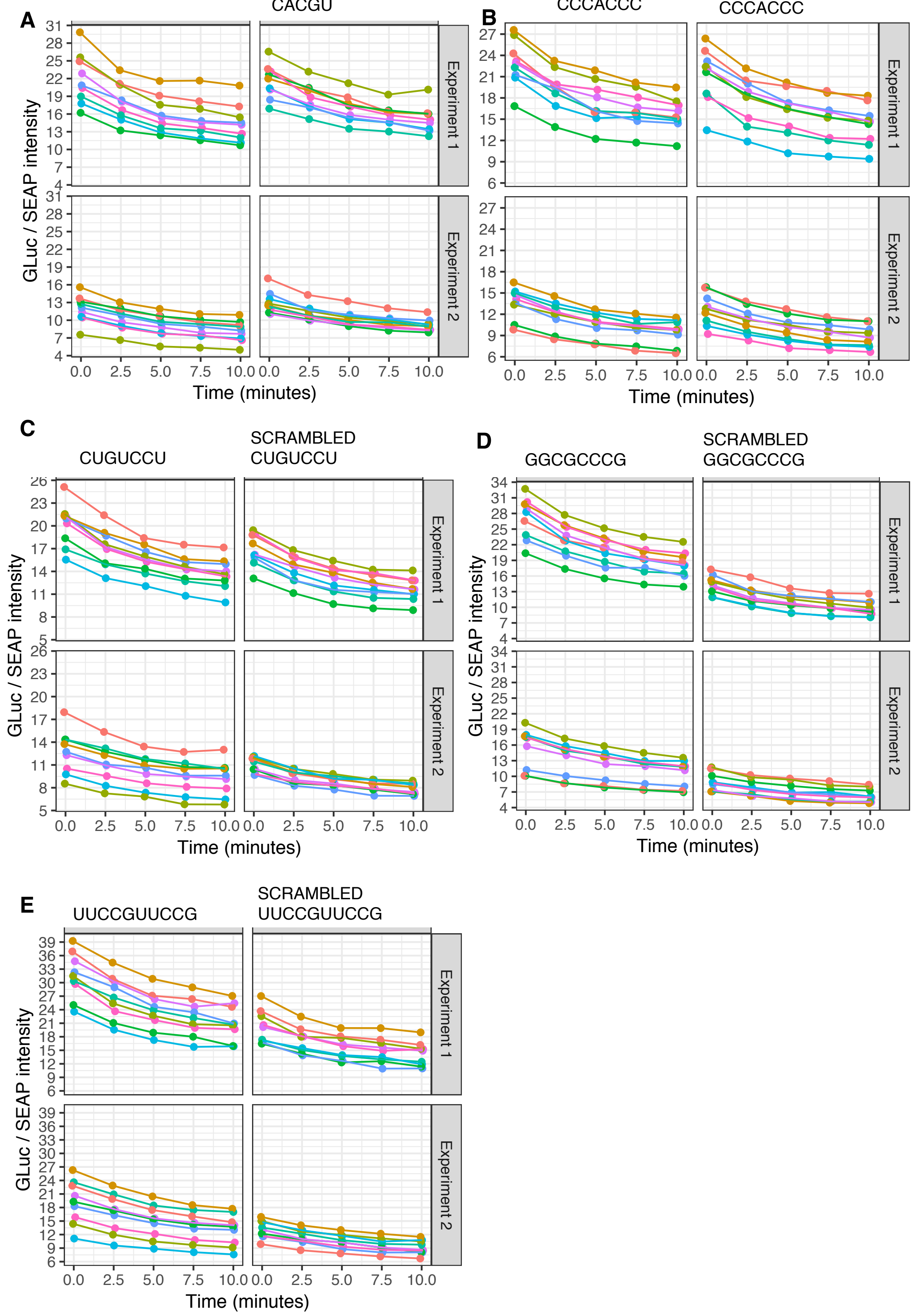

Figure S15: Time course Gluc/SEAP intensity values per 5' UTR motif and its scrambled version.

S16

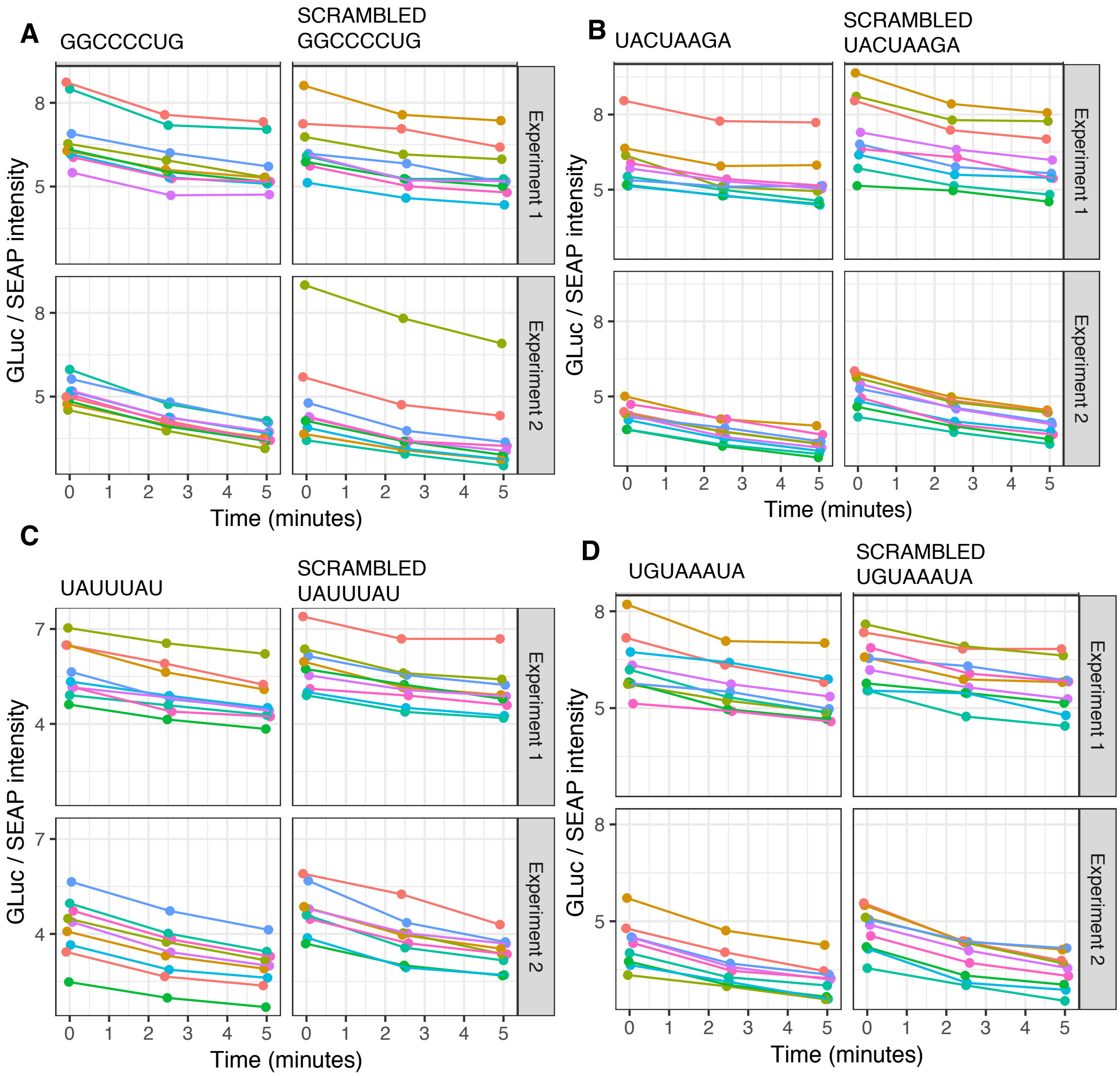

Figure S16: Time course Gluc/SEAP intensity values per 3' UTR motif and its scrambled version.

REFERENCES

Aberg K, Saetre P, Jareborg N & Jazin E (2006) Human QKI, a potential regulator of mRNA expression of human oligodendrocyte-related genes involved in schizophrenia. *Proc. Natl. Acad. Sci.* **103:** 7482–7487 Available at: http://www.ncbi.nlm.nih.gov/pubmed/16641098 [Accessed November 20, 2018]

Antonicka H, Sasarman F, Nishimura T, Paupe V & Shoubridge EA (2013) The Mitochondrial RNA-Binding Protein GRSF1 Localizes to RNA Granules and Is Required for Posttranscriptional Mitochondrial Gene Expression. *Cell Metab.* **17:** 386–398 Available at: http://www.ncbi.nlm.nih.gov/pubmed/23473033 [Accessed December 5, 2018]

FANTOM Consortium and the RIKEN PMI and CLST (DGT), Forrest ARR, Kawaji H, Rehli M, Baillie JK, de Hoon MJL, Haberle V, Lassmann T, Kulakovskiy I V, Lizio M, Itoh M, Andersson R, Mungall CJ, Meehan TF, Schmeier S, Bertin N, Jørgensen M, Dimont E, Arner E, Schmidl C, et al (2014) A promoter-level mammalian expression atlas. *Nature* **507:** 462–470 Available at: http://www.ncbi.nlm.nih.gov/pubmed/24670764 [Accessed December 5, 2018]

Filipovska A, Razif MFM, Nygård KKA & Rackham O (2011) A universal code for RNA recognition by PUF proteins. *Nat. Chem. Biol.* **7:** 425–427 Available at: http://www.ncbi.nlm.nih.gov/pubmed/21572425 [Accessed December 5, 2018]

Geuens T, Bouhy D & Timmerman V (2016) The hnRNP family: insights into their role in health and disease. *Hum. Genet.* **135:** 851–867 Available at: http://www.ncbi.nlm.nih.gov/pubmed/27215579 [Accessed December 5, 2018]

Kanhoush R, Beenders B, Perrin C, Moreau J, Bellini M & Penrad-Mobayed M (2010) Novel domains in the hnRNP G/RBMX protein with distinct roles in RNA binding and targeting nascent transcripts. *Nucleus* **1:** 109–122 Available at: http://www.ncbi.nlm.nih.gov/pubmed/21327109 [Accessed November 20, 2018]

Lizio M, Harshbarger J, Shimoji H, Severin J, Kasukawa T, Sahin S, Abugessaisa I, Fukuda S, Hori F, Ishikawa-Kato S, Mungall CJ, Arner E, Baillie J, Bertin N, Bono H, de Hoon M, Diehl AD, Dimont E, Freeman TC, Fujieda K, et al (2015) Gateways to the FANTOM5 promoter level mammalian expression atlas. *Genome Biol.* **16:** 22 Available at: http://www.ncbi.nlm.nih.gov/pubmed/25723102 [Accessed December 5, 2018]

Shetty S (2005) Regulation of urokinase receptor mRNA stability by hnRNP C in lung epithelial cells. *Mol. Cell. Biochem.* **272:** 107–18 Available at: http://www.ncbi.nlm.nih.gov/pubmed/16010978 [Accessed December 5, 2018]

Teplova M, Hafner M, Teplov D, Essig K, Tuschl T & Patel DJ (2013) Structure-function studies of STAR family Quaking proteins bound to their in vivo RNA target sites. *Genes Dev.* **27:** 928–940 Available at: http://www.ncbi.nlm.nih.gov/pubmed/23630077 [Accessed November 20, 2018]

Uhlen M, Fagerberg L, Hallstrom BM, Lindskog C, Oksvold P, Mardinoglu A, Sivertsson A, Kampf C, Sjostedt E, Asplund A, Olsson I, Edlund K, Lundberg E, Navani S, Szigyarto CA-K, Odeberg J, Djureinovic D, Takanen JO, Hober S, Alm T, et al (2015) Tissue-based map of the human proteome. *Science (80-.).* **347:** 1260419–1260419 Available at: http://www.ncbi.nlm.nih.gov/pubmed/25613900 [Accessed November 20, 2018]

Wang S, Morré DM & Morré DJ (2003) Sera from cancer patients contain two oscillating ECTO-NOX activities with different period lengths. *Cancer Lett.* **190:** 135–41 Available at: http://www.ncbi.nlm.nih.gov/pubmed/12565167 [Accessed December 5, 2018]
