# Supplementary material for: Quantification and discovery of sequence determinants of protein‐per‐mRNA amount in 29 human tissues
Source: Mol Syst Biol. 2019 Feb 18;15(2):e8513. doi: 10.15252/msb.20188513 (PMC6379048; doi:10.15252/msb.20188513)
Supplement: Supplementary file 2 — Expanded View Figures PDF [file MSB-15-e8513-s002.pdf]

Expanded View Figures

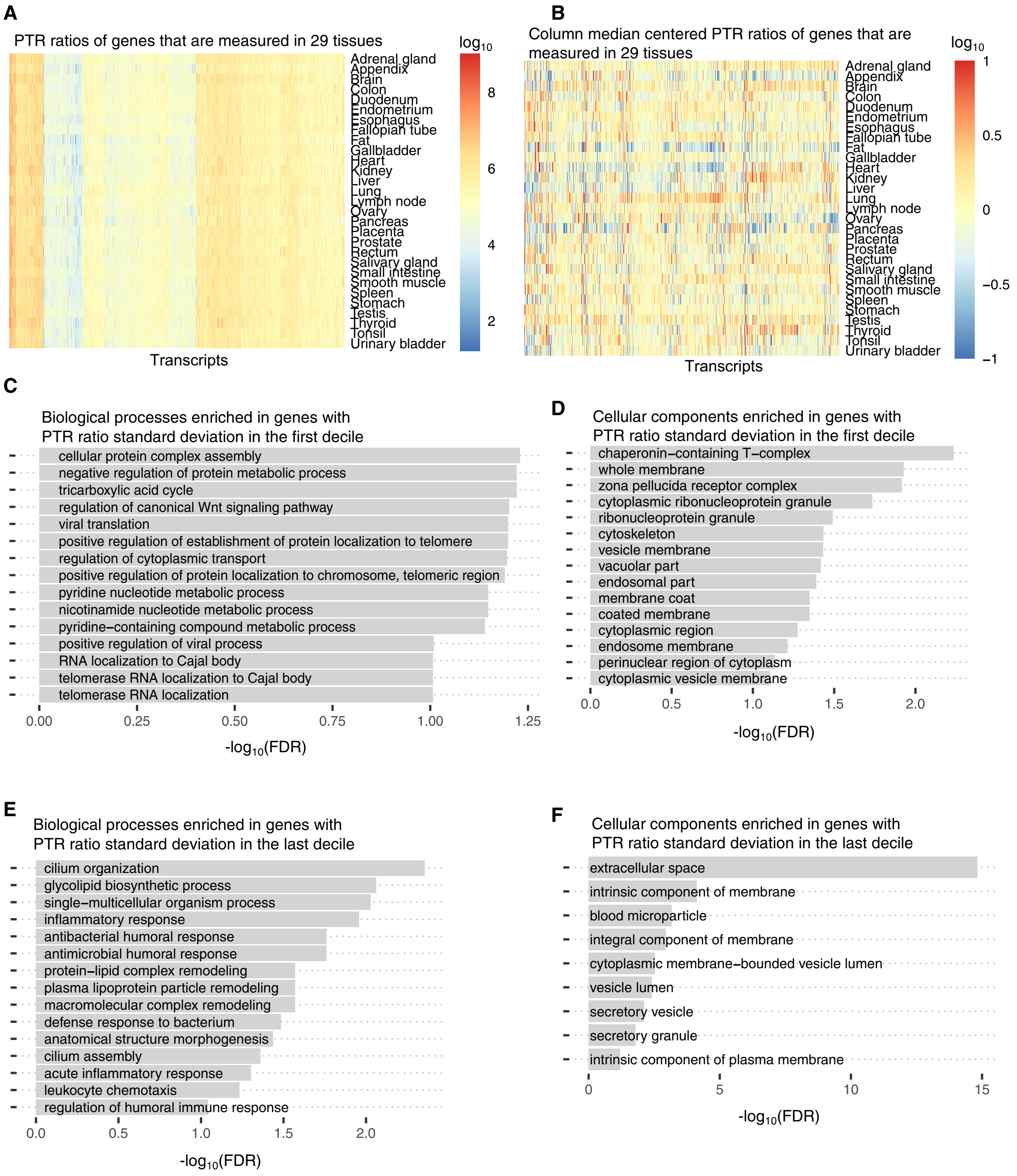

Figure EV1.

 © 2019 The Authors

Figure EV1. Variation of PTR ratio across tissues.

A PTR ratios of the 4,506 genes that are measured at the transcriptome level and at the proteome level in 29 tissues.
B PTR ratio fold-change per gene across tissues. Values shown in (A) are centered per gene based on their median values across tissues.
C Gene ontology terms (Biological Process) enriched for genes whose PTR standard deviation across tissues is in the first decile ((0.08,0.22] in $\log_{10}$) among 9,665 genes which are expressed in at least five tissues.
D Gene ontology terms (Cellular Component) enriched for genes whose PTR standard deviation across tissues is in the first decile ((0.08,0.22] in $\log_{10}$) among 9,665 genes which are expressed in at least five tissues.
E Gene ontology terms (Biological Process) enriched for genes whose PTR standard deviation across tissues is in the last decile ((0.7, 1.73] in $\log_{10}$) among 9,665 genes which are expressed in at least five tissues.
F Gene ontology terms (Cellular Component) enriched for genes whose PTR standard deviation across tissues is in the last decile ((0.7, 1.73] in $\log_{10}$) among 9,665 genes which are expressed in at least five tissues.

Figure EV2. Upstream AUG, start codon context, and stop codon context.

A Transcripts having at least one out-of-frame uAUG have significantly smaller PTR ratios across 29 tissues (corrected for other sequence elements) compared to transcripts with either no uAUG or only with in-frame uAUG(s) (Wilcoxon test, fold-change = 0.75, $P = 6.8 \times 10^{-17}$). Shown are the quartiles (boxes and horizontal lines).
B Transcripts with only one out-of-frame uAUG which is not followed by an in-frame stop codon are associated with 22% smaller PTR ratios (corrected for other sequence elements) compared to transcripts with only one out-of-frame uORF (Wilcoxon test, fold-change = 0.78, $P = 3.9 \times 10^{-2}$). Shown are the quartiles (boxes and horizontal lines).
C Average 100-vertebrate PhastCons score (*y*-axis, Materials and Methods) per position relative to the uAUG instances in 5′ UTR (*x*-axis). *P*-values assess significance of the average 100-vertebrate PhastCons scores at the motif sites compared to the two 10-nucleotide flanking regions (Materials and Methods).
D Median effect (dot) and range across 29 tissues (bar) of a single nucleotide mismatch relative to consensus sequence in a [−6, +6] nt window centered at first nucleotide of the canonical start codon (top). Position weight matrix logo showing information in bits (*y*-axis) computed across all 11,575 transcripts (bottom).
E Same as (D), centered at canonical stop codon.
F Transcripts with an opal (UGA) canonical stop codon have significantly smaller median PTR ratios across 29 tissues (corrected for other sequence elements) compared to transcripts with an ochre (UAA) or an amber (UAG) stop codon. Shown are the quartiles (boxes and horizontal lines), the furthest data points still within 1.5 times the interquartile range of the lower and upper quartiles (whiskers), *P*-values for two-sided Wilcoxon test (*P*), and fold-change (FC).
G Transcripts with a cytosine at the +1 position relative to the stop codon have significantly smaller median PTR ratios across 29 tissues (corrected for other sequence elements) independently of the stop codon type. Shown are the quartiles (boxes and horizontal lines) and furthest data points still within 1.5 times the interquartile range of the lower and upper quartiles (whiskers).

© 2019 The Authors

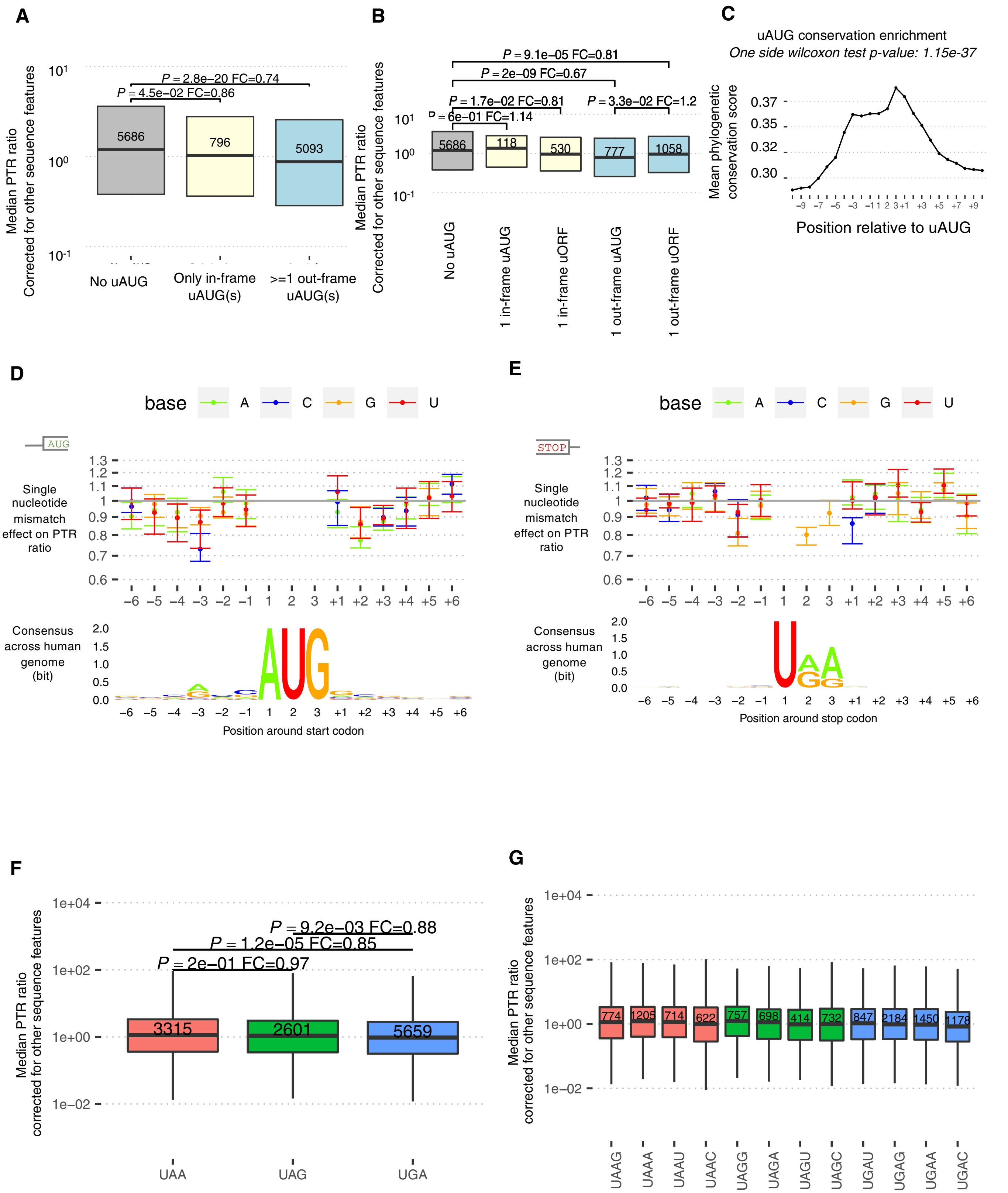

Figure EV2.

© 2019 The Authors

Figure EV3. 5' UTR k-mers and positional effects of codon on PTR ratio.

A First and third columns: Motif information content logos for the 25 k-mers of panel E in Fig 2 but AUG, obtained by motif consensus sequence search in 11,575 5′ UTR sequences allowing for one mismatch (Materials and Methods). Second and fourth columns: number and percentage of transcripts consensus motif sequence among the 11,575 transcripts (first line) and best significantly matching RNA binding protein motif of the database ATtRACT (Giudice *et al*, 2016) together with the ATtRACT motif quality score Q (value between 0 and 1, the higher the better; Materials and Methods).

B Log_2-transformed frequencies of 20 amino acids in the coding region explain on average 15% of the variance in tissue-specific PTR ratios (min 12%, max 17%). In comparison, log_2-transformed frequencies of 61 codons, which inherently encode for amino acid frequency and synonymous codon usage, explain on average 16% of the variance in PTR ratios (min 13%, max 20%).

C Relative codon frequencies per bin of 15 codons (columns). Codon frequency is differentially distributed in the 5′ end of the CDS (from codon 2 to codon 31) compared to the rest of the coding region.

D In spite of the distinctive codon frequency composition of the coding region 5′ end as displayed in (C), twofold codon frequency increase effect in the proximal 15 codons significantly correlates with the twofold codon frequency increase effect in the rest of the coding region (Spearman's correlation = 0.4, $P < 0.0024$; Materials and Methods).

© 2019 The Authors

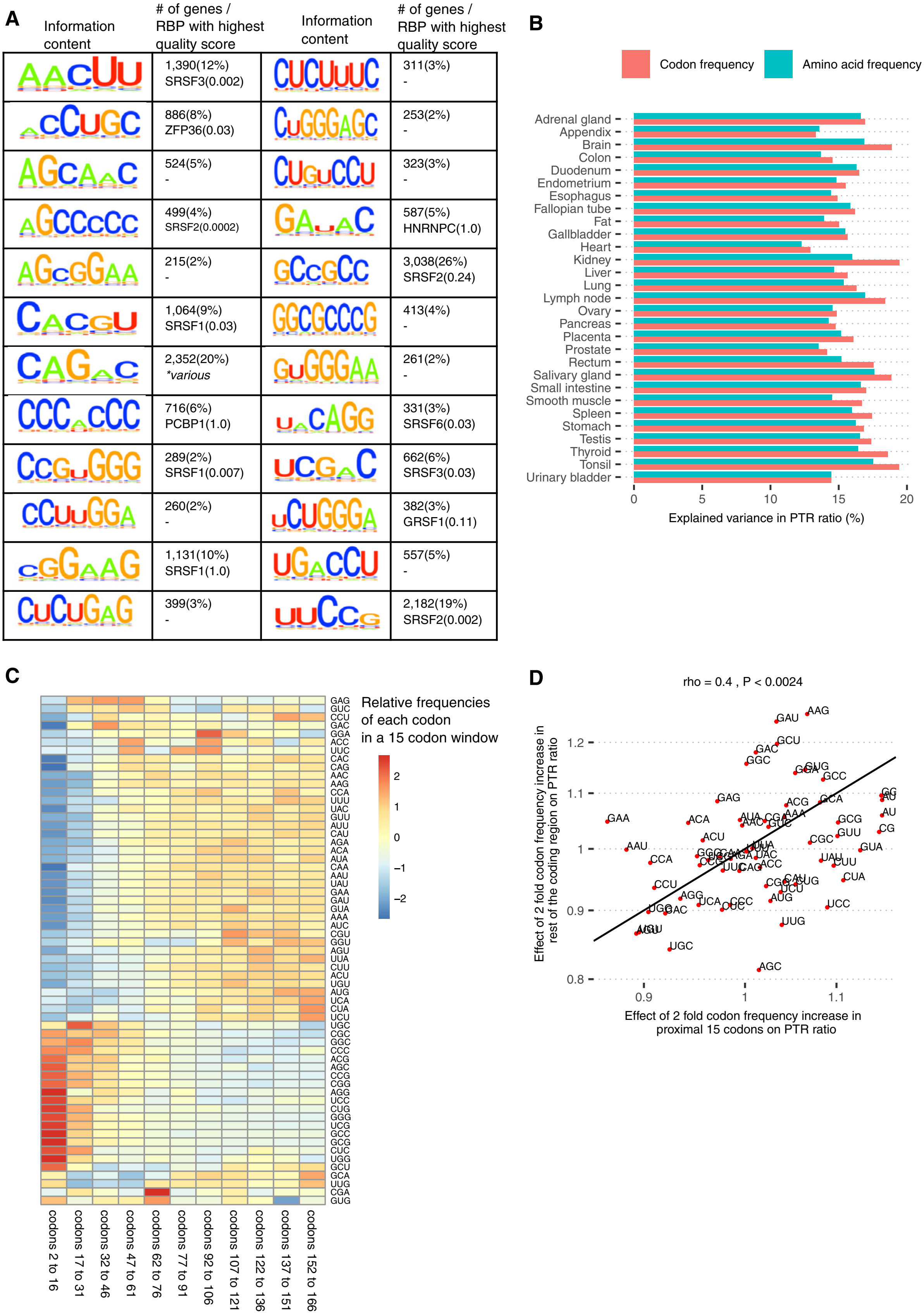

Figure EV3.

 © 2019 The Authors

Figure EV4. PTR-AI correlates with codon decoding time and codon effect on mRNA half-life.

A Median PTR-AI across tissues negatively correlates with expected codon decoding times in 17 ribosome profiling datasets (O'Connor et al, 2016).

B Median PTR-AI across tissues highly correlates with mRNA half-life fold-changes associated with twofold frequency increase of codons in K562 (Schwalb *et al*, 2016), HEK293 (Schueler *et al*, 2014), and HeLa Tet-off cells (Tani *et al*, 2012).

▶

© 2019 The Authors

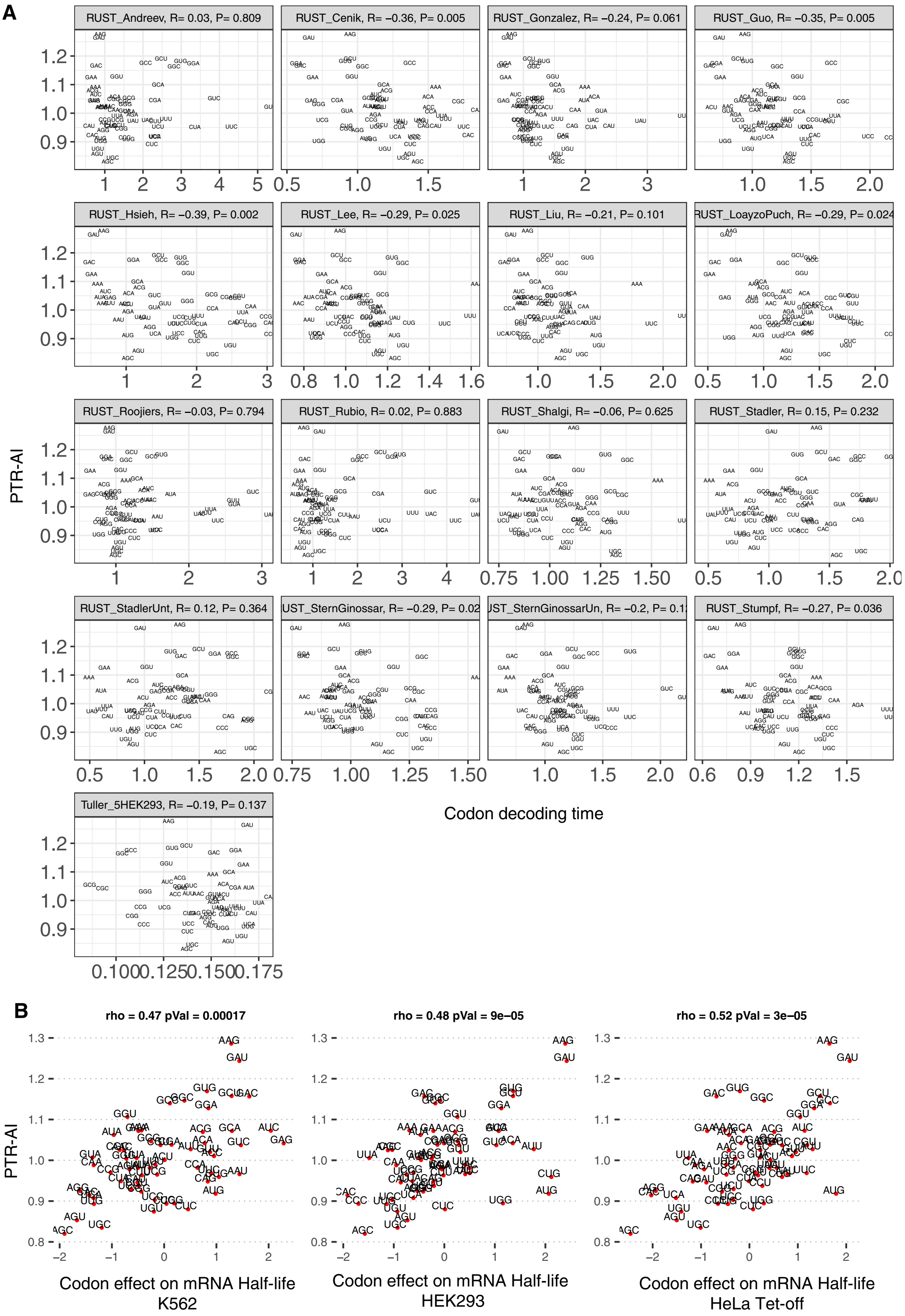

Figure EV4.

© 2019 The Authors

Figure EV5. Amino acid effects on PTR ratios and explained variance breakdown for PTR ratio, protein half-life, mRNA level, and mRNA half-life.

A Differences of amino acid effects on protein half-life (*x*-axis) significantly correlate with effects of single amino acid substitutions on protein thermodynamic stability (*y*-axis; Dehouck *et al*, 2009).

B Amino acid effect on protein half-life significantly correlates with the amino acid hydrophobicity value.

C Distributions of explained variances by linear models of individual sequence feature groups in PTR ratios of 29 tissues. The distribution of the total explained variance by the linear model that combines all of the listed sequence features is displayed in the first line. Shown are the quartiles (boxes and vertical lines) and furthest data points still within 1.5 times the interquartile range of the lower and upper quartiles (whiskers).

D Same as (C), with the difference that the response variable is set to be Ensembl Gene ID matched protein half-lives of HeLa cells (Zecha *et al*, 2018), B cells, NK cells, hepatocytes, and monocytes (Mathieson *et al*, 2018).

E Same as (C), with the difference that the response variable is set to be the Ensembl Transcript ID matched tissue-specific mRNA levels of 29 tissues.

F Same as (C), with the difference that the response variable is set to be Ensembl Transcript ID matched mRNA half-lives in K562 (Schwalb *et al*, 2016), HEK293 (Schueler *et al*, 2014), and HeLa Tet-off cells (Tani *et al*, 2012).

© 2019 The Authors

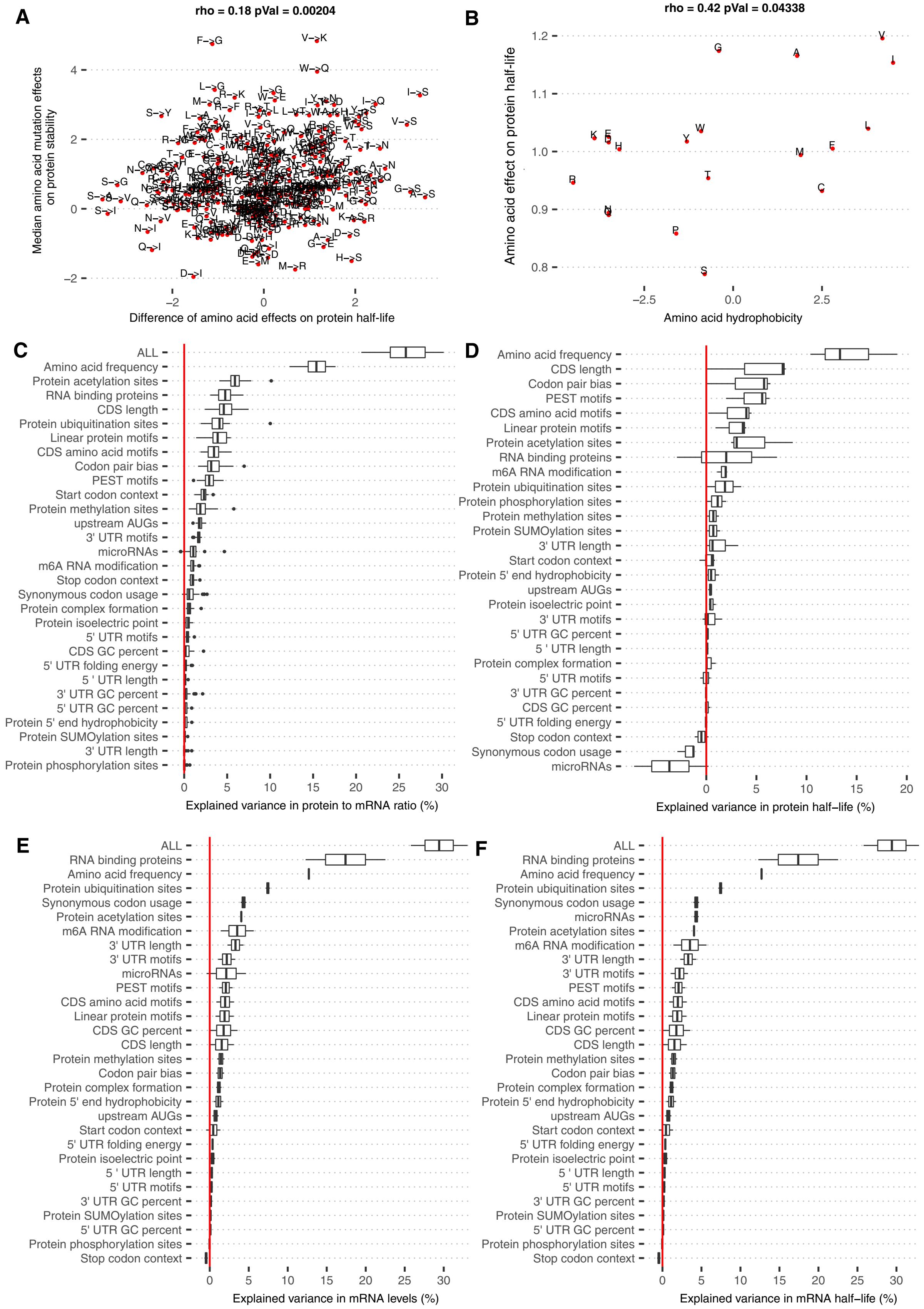

Figure EV5.

© 2019 The Authors
